# Trans-cellular tunnels induced by the fungal pathogen *Candida albicans* facilitate invasion through successive epithelial cells without host damage

**DOI:** 10.1038/s41467-022-31237-z

**Published:** 2022-06-30

**Authors:** Joy Lachat, Alice Pascault, Delphine Thibaut, Rémi Le Borgne, Jean-Marc Verbavatz, Allon Weiner

**Affiliations:** 1 Sorbonne Université, Inserm, CNRS, Centre d’Immunologie et des Maladies Infectieuses, Cimi-Paris, 75013 Paris, France; 2 Université Paris Cité, CNRS, Institut Jacques Monod, 75013 Paris, France

**Keywords:** Fungal pathogenesis, Cellular microbiology, Pathogens

## Abstract

The opportunistic fungal pathogen *Candida albicans* is normally commensal, residing in the mucosa of most healthy individuals. In susceptible hosts, its filamentous hyphal form can invade epithelial layers leading to superficial or severe systemic infection. Although invasion is mainly intracellular, it causes no apparent damage to host cells at early stages of infection. Here, we investigate *C. albicans* invasion in vitro using live-cell imaging and the damage-sensitive reporter galectin-3. Quantitative single cell analysis shows that invasion can result in host membrane breaching at different stages and host cell death, or in traversal of host cells without membrane breaching. Membrane labelling and three-dimensional ‘volume’ electron microscopy reveal that hyphae can traverse several host cells within trans-cellular tunnels that are progressively remodelled and may undergo ‘inflations’ linked to host glycogen stores. Thus, *C. albicans* early invasion of epithelial tissues can lead to either host membrane breaching or trans-cellular tunnelling.

## Abstract (web summary)

The fungal pathogen *Candida albicans* forms filamentous hyphae that can invade the epithelium. Here, Lachat et al. show that *C. albicans* early invasion of epithelial tissues can lead to either host membrane breaching and host cell death, or trans-cellular tunnelling through host cells without membrane breaching.

## Introduction

The human fungal pathogen *Candida albicans* colonizes the oral, genital and intestinal mucosa of most healthy individuals and is part of the normal commensal flora^1^. *C. albicans* often causes superficial infections such as oral or vaginal thrush, and in susceptible hosts can invade the gastrointestinal mucosa and enter the bloodstream, leading to a severe and life-threatening systemic infection^2^. *C. albicans* possesses a variety of virulence traits, allowing it to colonize within the microbiota when commensal, and invade host tissues during infection. *C. albicans’* ability to undergo a morphological switch, transitioning from round yeast cells to a filamentous hyphal form in response to various environmental cues, is considered to be its most important virulence trait, and has been strongly linked to its ability to invade and damage host tissue^3,4^.

Infection of epithelial layers proceeds through a series of sequential steps: attachment, initiation of hyphal growth, invasion and damage^5^. Attachment to the host cell surface is mediated by various adhesins, such as Als3, which binds to E-cadherin receptor on epithelial cells, as well as Hwp1, Eap1 and Iff4 among others^6–8^.

Invasion by *C. albicans* is thought to be mainly an intracellular (transcellular) process, at least until the loosening of epithelial connections and barrier breakdown^9–12^, as indicated by two-dimensional electron microscopy (EM) studies and differential staining invasion assays that suggest that invasion originates predominantly at the apical surface of epithelial cells^5,10,13–15^.

While invasion begins around one or two hours after attachment, host cell damage, measured by LDH or ^51^Cr release assays, has been reported to occur at a later stage, usually after 6–24 h, depending on the epithelial tissue^14–17^. Indeed, the major cause of damage during *C. albicans* epithelial infection is thought not to be invasion per se, but other factors such as candidalysin, a fungal peptide toxin secreted during infection^18^. Epithelial cells infected by a mutant deficient in secretion of candidalysin are still invaded normally, but suffer no apparent damage^11,18–20^. How *C. albicans* intracellular invasion can proceed without damaging host cells is currently not well understood^21^. While hyphal extension and the resulting invagination of the host plasma membrane into the so-called ‘invasion pocket’ can account for at least a portion of intracellular invasion, further hyphal extension within the host, and even more so into neighbouring host cells, is expected to lead to eventual host membrane breaching and damage due to increased membrane stretching^22,23^. Current models hypothesize the presence of a non-damaging intracellular invasion route, though the mechanism underlying such a route has not been described^11,20^.

Two mechanisms of *C. albicans* invasion are known: induced endocytosis and active penetration^20,24^. Induced endocytosis is considered a “host-driven” process, whereby invasins expressed on the surface of *C. albicans* hyphae induce host cells to produce pseudopods that engulf the pathogen and pull it inside the cell, a process analogous to the “trigger” mechanism employed by several invasive bacteria such as *Shigella flexneri* and *Salmonella enterica serovar Typhimurium*^14,15,25–27^. In epithelial cells, the invasins Als3 or Ssa1 activate an E-cadherin mediated, clathrin-dependent pathway, though an E-cadherin-independent pathway has been suggested as well^7,17,28–30^. Active penetration is considered a “fungal-driven” process that relies on a combination of physical forces exerted by extending hyphae and secreted fungal factors such as aspartyl proteinases (Saps), though still relatively little information about this mechanism exists^31,32^. The actin polymerization inhibitor Cytochalasin D has been reported to disrupt invasion by induced endocytosis but not by active penetration, marking a key distinction between these two mechanisms^14,27,30^. While oral, vaginal and microfold (M) cells are invaded via both mechanisms, enterocytes are invaded only via active penetration, though the underlying reason for this cell type dependency is unclear^9,14,33–35^. On the host side the response to invasion remains poorly described, though actin, clathrin, dynamin and cortactin have been shown to be recruited to the site of invasion and to have a functional role in *C. albicans* internalization in HEK293 and JEG-3 cells^29,36^. The small GTPases Rac1, Cdc42 and Rho were also shown to be co-localized with actin at the site of invasion, though their precise function in this context is not known^37^.

In general, two strategies can be employed by invasive pathogens to enter host cells: breaching of host membranes and entry into the host cytosol or residence within a membrane-bound compartment. This distinction between cellular “niches” has been especially useful for describing the intracellular lifestyles of invasive bacteria which include: membrane breaching pathogens like *S. flexneri, Rickettsia spp*. and *Listeria monocytogenes*; pathogens that reside in membrane-bound compartments like *Brucella spp., Legionella pneumophila* and *Chlamydia spp.;* and pathogens that can adapt both lifestyles like *S. enterica* and *Mycobacterium tuberculosis*^25,38–40^. The fungal pathogen *Cryptococcus neoformans* resides within a membrane-bound compartment in macrophages, where it can induce non-lytic expulsion through fusion of the phagosome and plasma membranes^41^. In contrast, the invasive lifestyle of *C. albicans* is still poorly understood, with limited information derived mostly from end-point assays and two-dimensional EM regarding the host cellular niches encountered during epithelial invasion^10,13–15,27^. Indeed, it is currently unclear if and when host membranes are breached during *C. albicans* invasion; a key aspect in understanding this pathogen’s invasive lifestyle.

In this work we investigate the early invasive lifestyle of *C. albicans* during in vitro epithelial infection using live cell imaging combined with the real-time damage sensitive reporter galectin-3. We provide a quantitative account in both HeLa and Caco-2 cells of host membrane breaching events occurring at different stages of invasion, revealing two distinct invasive lifestyles: damaging and non-damaging. Using three-dimensional “volume” electron microscopy and host membrane labelling, we go on to show that non-damaging invasion is mediated by host membrane-derived trans-cellular tunnels that can extend through several host cells, and are progressively remodelled with each invaded cell. Our work sheds light on long standing questions regarding the precise nature of invasion and host damage during the early stages of *C. albicans* infection of epithelial layers.

## Results

### New experimental pipeline combines the galectin-3 damage sensitive reporter and live-cell imaging to study *C. albicans* invasion

**Fig. 1:**
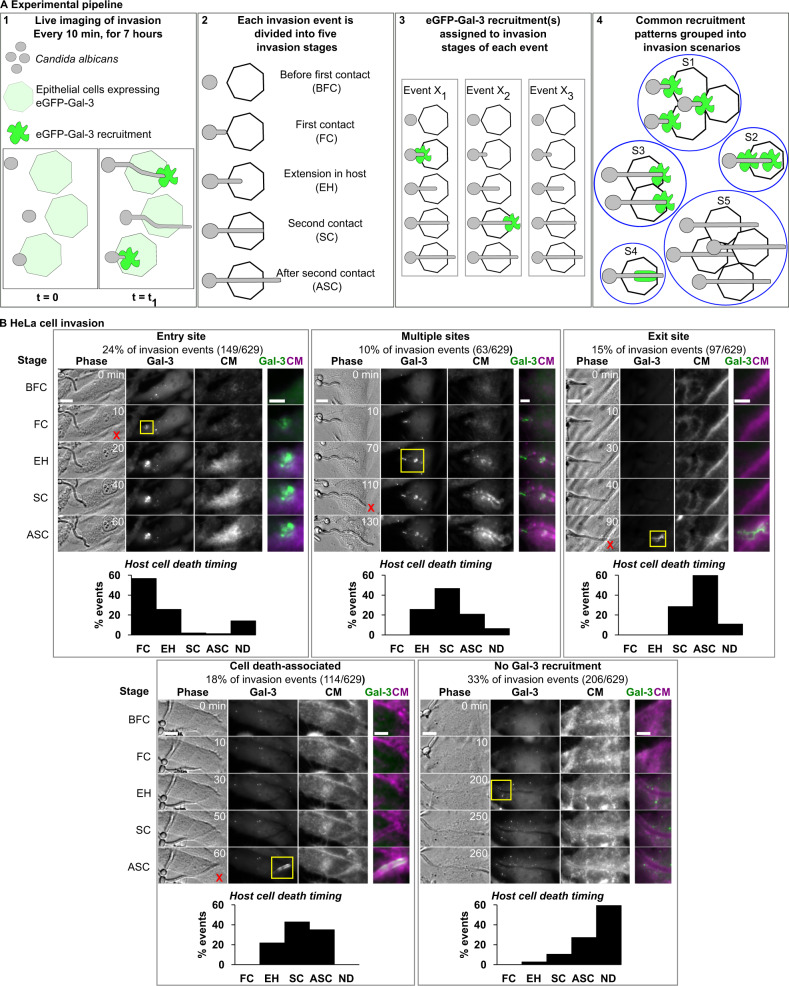
*C. albicans* epithelial cell invasion studied using eGFP-Galectin-3 damage sensitive reporter and live-cell imaging. The experimental pipeline (**A**) and results for HeLa invasion (**B**) are presented. (A1) *C. albicans* invasion of epithelial cells stably expressing eGFP-Galectin-3 is studied using live-cell imaging. Localized high-intensity eGFP-Galectin-3 recruitments recorded are indicative of host membrane damage. (A2) Each invasion event, defined as a single interaction between a *C. albicans* hypha and a host cell over time, is sub-divided into five invasion stages: before first contact with host plasma membrane (BFC), first contact with host plasma membrane (FC), hypha extension within the host (EH), second contact with the host plasma membrane (SC), after second contact with the host plasma membrane (ASC). (A3) For every invasion event (X_1_, X_2_..), eGFP-Galectin-3 recruitments are assigned to the stage in which they occurred. (A4) All invasion events sharing common eGFP-Galectin-3 recruitment patterns are grouped into invasion scenarios (S_1_, S_2_..). **B** *C. albicans* HeLa invasion scenarios (*n* = 629 invasion events). For each scenario a representative invasion event is presented, divided into the five invasion stages described in (**A**). For each stage a single section is presented in phase, Gal-3, and CellMask (CM) channels, as well as an inset (yellow frame) containing a composite view of the Gal-3 (green) and CM (magenta) channels. Cell death (if it occurred) is marked with a red ‘x’ in the corresponding invasion stage. The distribution of cell death timing as a function of the invasion stage is presented for each scenario. ND stands for no cell death during invasion. Scale bars are 10 µm or 5 µm in insets. Source data are provided as a Source Data file.

We developed a new experimental pipeline to study membrane breaching events during *C. albicans* epithelial cell invasion at the single cell level using the damage sensitive reporter galectin-3 combined with multi-dimensional live-cell imaging and other cell markers. Galectin-3 is a small soluble protein localized in the cytosol, which has affinity to β-galactose-containing carbohydrates. These moieties are typically found on the luminal side of intracellular vesicles and at the outer leaflet of the plasma membrane, but not in the cytosol nor the nucleus^42^. By expressing galectin-3 linked to a fluorescent reporter, the precise location and timing of membrane breaching events large enough to allow galectin recruitment can be tracked in real-time, as upon damage fluorescent galectin-3 flows from the cytosol, through the ruptured membrane and onto the cell surface, where it binds to moieties not normally accessible to it^42,43^. Galectin-3 has been used to study the lifestyles of invasive bacteria such as *S. flexneri* and *S. enterica*^39,42,44–46^, as well as *C. albicans* escape from macrophages^47^. In short, the experimental pipeline consists of the following steps (see Fig. 1A): (1) *C. albicans* is cultured to log phase and diluted to a multiplicity of infection (MOI) of 0.1. In parallel, epithelial cells stably expressing eGFP-galectin-3 (from here on referred to as ‘Gal-3’) are cultured to confluency. The cell lines used in this study were verified to not overexpress eGFP-galectin-3 compared to endogenous galectin-3 via Western blot and immunoprecipitation (see Supplementary Fig. 1). Next, host cells are stained with CellMask (CM), a membrane stain suitable for live-cell imaging which labels the entire host plasma membrane and endocytic compartment. Importantly, CM staining is performed for 10 min followed by a wash before the addition of *C. albicans*, such that *C. albicans* membranes are not labelled by CM at any point during the experimental pipeline.

Next, *C. albicans* is added above the epithelial layer, followed by live-cell imaging of multi-channel z-stacks acquired every 10 min for 7 h.

(2) Post-acquisition, every invasion event, defined as an interaction between a single *C. albicans* hypha and a single host cell, is identified and analysed individually. Each event is sub-divided into five invasion stages: 1. Before first contact with the host plasma membrane (BFC), 2. First contact with the host plasma membrane (FC), 3. Extension within the host cell (EH), 4. Second contact with host plasma membrane (SC), 5. After second contact with the host plasma membrane (ASC).

(3) Observed Gal-3 recruitments are assigned to their corresponding invasion stage.

(4) All invasion events presenting common stage-dependant Gal-3 recruitment patterns are grouped into invasion scenarios that represent the different ways in which host membrane damage (or lack thereof) is manifested during *C. albicans* invasion into a specific cell line. During the analysis, the localization patterns of CM and host cell death timing (determined by membrane blebbing and condensed nuclei observed in phase contrast or by propidium iodide staining^48^) are also recorded and assigned to their corresponding invasion stage for every invasion event. However, only the Gal-3 signal is used to define the different invasion scenarios (for full pipeline details see Materials and Methods).

### Invasion into HeLa cells occurs via five invasion scenarios

We first studied *C. albicans* invasion into HeLa cells (from here on referred to as HeLa) expressing Gal-3, as this non-polar cell line has been previously used to characterize in detail host membrane damage and the invasive lifestyles of *S. flexneri* and *S. enterica*^39,45^. HeLa have also been used to study *C. albicans* invasion, with both induced endocytosis and active penetration implicated as the mechanisms of invasion^26,29,30,49^. Wild-type prototrophic *C. albicans* strain BWP17 (WT strain)^50^ invasion into HeLa expressing Gal-3 was studied using the experimental pipeline described above. Overall, we studied 629 invasion events in six statistically comparable experiments, in a time frame of 0–7 h post infection.

Analysis of invasion events revealed five distinct invasion scenarios (see Fig. 1B, Supplementary Movies 1–5): ‘Entry site’ scenario: Gal-3 is recruited at the site of contact between hyphae and the host plasma membrane during FC (24% of events); ‘Multiple sites’ scenario: Gal-3 is recruited at multiple sites during FC and EH (10% of events); ‘Exit site’ scenario: Gal-3 is recruited at the second contact with the host plasma membrane during SC or ASC (15% of events); ‘Cell death-associated’ scenario: Gal-3 is recruited in an elongated pattern along the hypha trajectory. This recruitment pattern always coincided with host cell death, as revealed by cell death timing data (see details below) (18% of events); ‘No Gal-3 recruitment’ scenario: no Gal-3 recruitment is observed during invasion (33% of events). Overall, we observe that ‘damaging scenarios’ exhibiting Gal-3 recruitments constitute 67% of invasion events, with the ‘non-damaging’ ‘no Gal-3 recruitment’ scenario constituting the rest.

We calculated the host cell traversal time, defined as the time it takes for a hypha to extend from the FC stage to the SC stage, if no host cell death occurs. In HeLa, traversal time averages 70 min ± 38 min independently of the invasion scenario, suggesting host membrane breaching does not impact the overall dynamics of invasion (see Supplementary Fig. 2A). In order to confirm that Gal-3 recruitments were associated with internalized hyphae extending inside host cells, we used a fixed differential staining assay adapted from previous invasion studies^14,27^ (see Supplementary Fig. 3). Of note, while invading hyphae were often observed extending in proximity to or alongside host cell nuclei, *C. albicans* invasion into the nucleus was never observed.

The distribution of host cell deaths as a function of invasion stage was calculated for every invasion scenario based on phase contrast as described above (see Fig. 1B). Invasion was considered to have occurred without host cell death if at least 90 min after the SC stage no host cell death was observed (annotated as ND in Fig. 1B). During ‘damaging scenarios’ host cell death was observed in 92% of events, compared to 41% of events in the ‘no Gal-3 recruitment’ scenario. In the latter scenario, only 13% of all events resulted in host cell deaths during the FC, EH and SC stages, compared to 65% of events resulting in host cell deaths at these stages in the ‘damaging scenarios’. The elongated Gal-3 recruitment pattern observed in the ‘cell death-associated’ scenario always coincided with host cell death, within the temporal resolution of the experiment. This recruitment pattern is likely a result of general host cell membrane permeabilization allowing Gal-3 to bind membranes surrounding the hyphae’s path (see below). In order to corroborate our observations of host cell death distribution, we repeated HeLa cell invasion experiments in the presence of the cell death marker propidium iodide (PI) (*n* = 358 invasion events, see Supplementary Fig. 4). Host cell death distribution within the different invasion scenarios confirmed our initial observations, with host cell death observed during ‘damaging scenarios’ in 94% of events, compared to 69% of events in the ‘no Gal-3 recruitment’ scenario. In the latter scenario, only 26% of all events resulted in host cell deaths during the FC, EH and SC stages, compared to 61% of events resulting in host cell deaths at these stages in the ‘damaging scenarios’. The overall increase in host cell death detected using PI may be due to improved host cell death identification during live-cell imaging. Taken together, host membrane breaching during HeLa invasion is most often associated with host cell death, while invasion without host membrane breaching is most often associated with host cell survival, particularly during host cell traversal (FC, EH, SC stages).

As most studies thus far of damage inflicted during *C. albicans* in vitro infection have relied on population level detection of damage via LDH release assay, we monitored LDH release at 3-, 6- and 24-h post-infection of HeLa (see Supplementary Table 1)^11,14–16,30,32^. While LDH release was not detected at 6 h post infection using a MOI of 0.1 (used in our live cell imaging pipeline), it was detected using a MOI of 1 at this time point. This difference is likely due to lower sensitivity of the LDH release assay in detecting cell damage compared to its detection via Gal-3 at the single cell level. At 24 h post infection, cell damage was detected at both MOIs. Overall, these results are consistent with our observation of host cell damage inflicted during the early stages of HeLa cell infection.

Analysis of the host plasma membrane and endocytic compartment marker CM revealed a faint but clear uniform labelling of host cell membranes along the path of invasion during the ‘exit site’, ‘cell death-associated’ and the ‘no Gal-3 recruitment’ scenarios. In contrast, in the ‘entry site’ scenario, CM labelling was found only at the site of membrane breaching, and in the ‘multiple sites’ scenario, CM labelling was in the form of discontinuous puncta and patches along the path of invasion.

Finally, as induced endocytosis, but not active penetration, is inhibited by the actin polymerization inhibitor cytochalasin D, we examined the effect of cytochalasin D treatment on HeLa invasion using our experimental pipeline. Cytochalasin D treatment did not significantly alter the number of invasion events and the distribution of HeLa invasion scenarios was also not significantly altered (see Supplementary Fig. 5A). This result suggests that invasion in this cell type does not occur via induced endocytosis according to this distinguishing criterion, in agreement with previously published data^51^. However, another study in which endocytosis was directly monitored did determine a role for endocytosis in HeLa invasion^29^. Thus, as we did not directly monitor endocytosis in our study, a role for endocytosis in invasion in this cell type cannot be excluded.

**Fig. 2:**
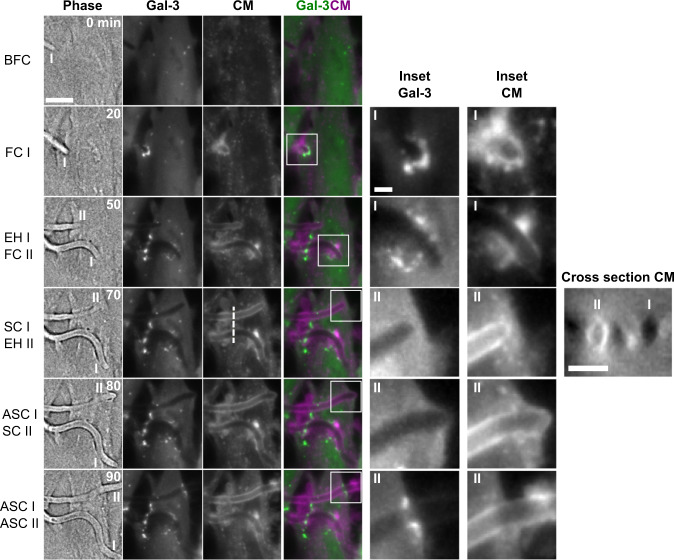
High-resolution live-cell imaging of two *C. albicans* hyphae invading a single HeLa cell via a ‘multiple sites’ scenario and an ‘exit site’ scenario. Six time points are presented, with the invasion stages defined in Fig. 1 corresponding to either filament I (‘multiple sites’ scenario) or II (‘exit site’ scenario). For each time point a phase image is presented together with a maximum intensity projection of three z-sections in the Gal-3 and CellMask (CM) channels, and a composite image showing Gal-3 (green) and CM (magenta) together. Two insets (Gal-3 and CM) are presented for each timepoint, corresponding to filament I or II. A cross-section in the CM channel through both filaments (dotted line) is presented at time point 70 min. A circular CM organization is observed around filament II but not filament I. No host cell death was observed during the course of live-cell imaging. Scale bars are 10 µm, 2 µm (inset) and 5 µm (cross-section). For other invasion scenarios see Supplementary Fig. 6.

Next, we used our experimental pipeline to study the three-dimensional dynamics of CM recruitment in detail. In one striking example, a single HeLa cell was invaded by two hyphae, each via a different invasion scenario (see Fig. 2, Supplementary Movie 6). Invasion by the first hypha (I) started with a Gal-3 recruitment during the FC stage, followed by smaller Gal-3 recruitments during the EH stage, indicating a ‘multiple sites’ invasion scenario. Around this hypha, only localized, discontinuous CM recruitment was observed along the path of invasion. Invasion by the second hypha (II) did not trigger Gal-3 recruitment until the ASC stage. This hypha was tightly surrounded by a continuous CM label throughout invasion. During the SC stage, a clear host plasma membrane outward deformation was observed, followed by a ring-like Gal-3 recruitment during the ASC stage, indicating an ‘exit site’ invasion scenario. A cross-section view of the CM signal through both hyphae shows the absence of CM around hypha I, and a circular CM organization around hypha II. These CM recruitment patterns were representative of all ‘multiple sites’ and ‘exit site’ scenarios studied. Overall, we observe that CM organization during HeLa invasion is dependent on the invasion scenario, with a continuous circular organization around invading hyphae in the ‘no Gal-3 recruitment’, ‘exit site’ and ‘cell-death associated’ scenarios, a patchy and discontinuous organization along the path of invasion in the ‘multiple sites’ scenario, and a localized recruitment at the site of membrane breaching in the ‘entry site’ scenario (see Supplementary Fig. 6). Interestingly, in sample regions where HeLa were not entirely confluent, we often observed a continuous CM label tightly enveloping ASC stage hyphae extending into the space between cells, suggesting host membranes can be stretched outwards by extending hyphae after invasion (see Supplementary Fig. 7).

### Invasion into Caco-2 cells occurs via a single non-damaging invasion scenario

The Caco-2 cell line is a widely used model for enterocytes and is of particular relevance to *C. albicans* invasion, as the gut is considered to be the reservoir for severe systemic infection^52,53^. Several studies have indicated that *C. albicans* invasion into polarized Caco-2 cells (from here on referred to as Caco-2) occurs only via active penetration^9,14,34^. No host damage (measured by LDH release) was detected in the first hours of Caco-2 infection, despite extensive invasion already taking place, suggesting that early infection in this cell line is non-damaging^11,14,16^.

**Fig. 3:**
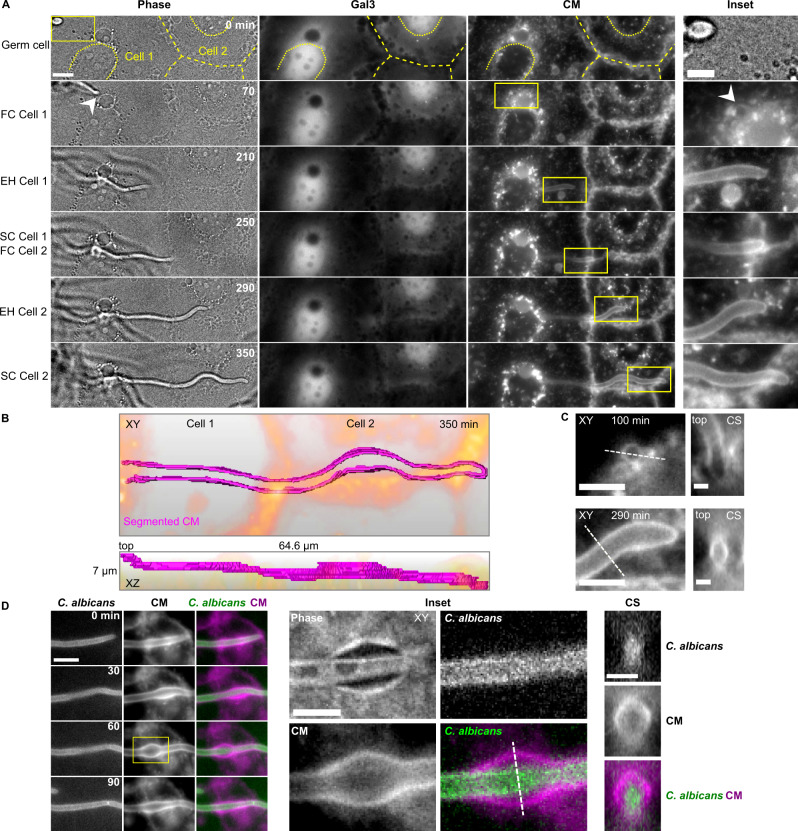
Invasion into Caco-2 occurs via a single non-damaging scenario. **A** Caco-2 expressing Gal-3 are invaded by a single invasion scenario without Gal-3 recruitment or host cell death. A representative live-cell imaging acquisition of invasions through two successive cells (cell 1 and cell 2) is presented. The six time points correspond to the invasion stages defined in Fig. 1. Phase (single z slice), Gal-3 and CM (maximum intensity projection of seven slices) channels, together with an inset (yellow frame) are presented for each time point. The outlines of the two invaded Caco-2 and their nuclei are highlighted with dashed and dotted yellow lines respectively. The entry point into the Caco-2 layer is marked by an arrowhead. **B** Three-dimensional representation of CM labelling (magenta) presented in (**A**) within the entire acquisition volume at a single time point (350 min). Epithelial cell outlines are in orange. A top view (XY) and side view (XZ) are presented together with the volume dimensions. **C** CM channel insets of two time points from the data presented in (A) are presented together with cross-section (CS) views derived from the dashed lines. **D** Live-cell imaging of infection of Caco-2 not expressing Gal-3 with a *C. albicans* strain expressing GFPγ. An average intensity projection of five slices is presented. A local transient ‘inflation’ is observed in the CM channel. An inset (yellow frame) showing a clear separation between the CM labelling and *C. albicans* when the ‘inflation’ is at its maximum size is presented in XY and in a cross section (CS) derived from the dotted line. As the hypha presented has extended from a previously invaded host cell, it is already entirely surrounded by CM labelling at time 0 min. Scale bars are 10 µm in (**A**, **D**), 5 µm in (**A**) Inset, (**C**) XY views and (**D**) inset, and 2 µm in (**C**) CS views.

Invasion into the Caco-2 clone C2BBe1 stably expressing Gal-3 was studied in 153 invasion events in three independent experiments, using the same experimental pipeline described above, in a time frame of 2–9 h post infection. Caco-2 polarization was confirmed by the presence of microvilli observed by EM (see below)^54^. In contrast to HeLa invasion, invasion into Caco-2 occurred via a single invasion scenario in which no detectable Gal-3 recruitment and no host cell death (detected by phase contrast) took place during live-cell imaging (see Fig. 3A, Supplementary Movie 7). In all invasion events, continuous CM labelling along the entire path of invading hyphae was observed. Interestingly, when several host cells were invaded in sequence, CM labelling appeared to be continuous, with no detectable gaps during the transition between cells (see Fig. 3A, B, Supplementary Movie 8). Cross-section views revealed a circular CM organization around invading hyphae, which appeared identical to the CM labelling observed during three of the HeLa invasion scenarios (‘no Gal-3 recruitment’, ‘exit site’ and ‘cell-death associated’). This circular organization could also be detected in a head-on view during early invasion when hyphae were nearly aligned along the microscopical z-axis (see Fig. 3C).

The host cell traversal time during Caco-2 invasion was 97 ± 54 min (see Supplementary Fig. 2B) and hyphae internalization was confirmed using a fixed differential staining assay (see Supplementary Fig. 3). As in HeLa, nuclear invasion was never observed in Caco-2.

Host cell damage and death during Caco-2 infection were further studied using live cell imaging of Caco-2 expressing Gal-3 in the presence of PI at early (2–9 h post infection) and late (19–29 h post infection) time points (see Supplementary Fig. 8). While no Gal-3 recruitment nor host cell death were detected at early time points, Gal-3 recruitment and host cell death were abundant at later time points, demonstrating that non-damaging invasion in Caco-2 is a phenomenon of early time points of infection. The absence of host damage in the first hours of Caco-2 infection was further confirmed via LDH release assay, in which host damage was detected at 24 h post infection but not at early time points of infection, in agreement with published data (see Supplementary Table 1)^11,14,16^.

In 25% of invasion events (*n* = 38/153) we observed a localized and transient expansion of the CM labelling surrounding invading hyphae. In order to visualize these membrane ‘inflations’ in detail, we performed invasion experiments into Caco-2 (not expressing Gal-3), using a *C. albicans* strain expressing GFPγ at the plasma membrane, allowing clear distinction between the CM signal and the hypha outline (see Fig. 3D, Supplementary Movie 9). Each inflation originated from the typical tight CM labelling around extending hyphae, then grew bigger over time until a clear separation between the CM signal and the hypha was detectable (see Fig. 3D inset), followed by a return to a tight CM labelling around the extending hypha. Inflations were observed along the entire path of invasion through Caco-2, and were never observed during HeLa invasion.

Finally, as for HeLa invasion, we examined the effect of cytochalasin D treatment on Caco-2 invasion using our experimental pipeline (see Supplementary Fig. 5B). We found that invasion into treated Caco-2 was unchanged relative to untreated cells and occurred via a single non-damaging invasion scenario. Treatment did not significantly alter the number of invasion events, suggesting that invasion in this cell type does not occur via induced endocytosis according to this distinguishing criterion, in agreement with previously published data^14^.

### Serial block face-scanning electron microscopy (SBF-SEM) of Caco-2 invasion provides a three-dimensional nano-scale view of hyphae invading successive host cells

**Fig. 4:**
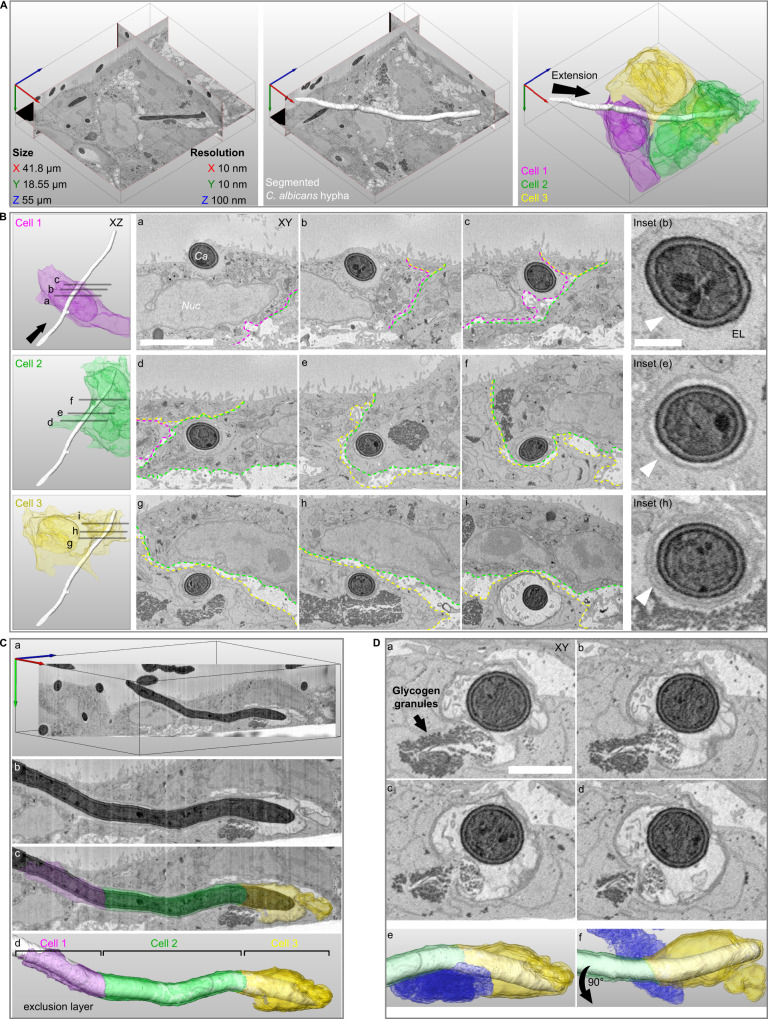
Serial block face-scanning electron microscopy (SBF-SEM) of Caco-2 invasion. **A** A representative SBF-SEM volume is presented together with dataset size and resolution in each axis (left panel). A *C. albicans* hypha in the process of invasion is visualized in three-dimensions (white, middle panel). Three invaded host cells are colour coded according to the sequence of invasion, cell 1 (magenta), cell 2 (green), cell 3 (yellow). The direction of filament extension is noted (arrow, right panel). **B** The organization of host membranes around the hypha is presented for each invaded cell with three cross-section views (Cell 1- a, b, c; Cell 2- d, e, f; Cell 3- g, h, i). The direction of filament extension is noted (arrow). The borders between cells are annotated according to the colours in (A) and the hypha (Ca) and the host nucleus (Nuc) are noted in (a). An inset from the middle cross-section in each cell is presented with arrowheads pointing to host membranes surrounding the hypha. The hypha in cell 1 is tightly enveloped by a thin membrane which in turn is surrounded by an ‘exclusion layer’ (EL) devoid of cellular structures (a, b, c, inset (b)). In cell 2, a thicker membrane enveloping a narrow lumen and the hypha is observed (d, e, f, inset (e)). In cell 3, two membranes surrounding a narrow lumen and the hypha are observed in (g, h, inset (h)). An ‘inflation’ containing multiple vesicles is observed in (i). **C** A cross-section through the hypha long-axis is presented, showing the continuous membrane structures across three cells (a, b). The ‘exclusion layer’ and membranes surrounding the hypha are visualized according to the invaded cell sequence (c, d). **D** A sequence of four sequential sections showing direct contact between the ‘inflation’ lumen and host glycogen granules (a–d). Glycogen granules are noted in (a). A visualization presented in the same orientation as in (**C**) shows the contact between the host glycogen store (blue) and the ‘inflation’ in cell 3 (e). A 90-degree rotated view is presented in (f). Scale bars are 5 µm in **B**, 1 µm in **B** inset and 2 µm in **D**.

Understanding precisely how *C. albicans* invades host cells without damage requires imaging at a resolution beyond the limit of standard light microscopy. Moreover, studying cellular structures that extend over long cellular distances and even several cells requires three-dimensional nano-scale imaging of large sample volumes; a combination difficult to obtain using traditional EM approaches^55^. We therefore studied invasion using serial block face-scanning electron microscopy (SBF-SEM), an emerging “volume” EM technique capable of imaging entire *C. albicans* filaments invading multiple host cells within a single 3D dataset at nano-scale resolution^56,57^ (see Fig. 4). As invasion into Caco-2 occurs via a single non-damaging invasion scenario, we targeted this cell line for SBF-SEM. Caco-2 were infected by WT *C. albicans* for 6 h, followed by EM sample preparation, which resulted in excellent sample contrast for both *C. albicans* internal structures like the Spitzenkörper and for membranes and organelles of the host^58^ (see Materials and Methods). Host cells presented clear microvilli at their apical surface, indicating monolayer polarization. Datasets were acquired in three different sessions, with a resolution of 10 nm in the acquisition plane and an axial resolution of 100 nm. Overall, 11 volumes were acquired containing 11 invasion sites in which the entire or nearly the entire invading hypha and the sequence of invaded host cells were captured, and an additional 30 invasion sites in which only a partial segment of an invading hypha was captured. A representative dataset containing a *C. albicans* filament invading three host cells in succession is presented in detail (see Fig. 4A, Supplementary Movie 10). A quantification of the features found in the 11 volumes containing complete or nearly complete invasion sites can be found in Supplementary Table 2.

### Invading filaments are enveloped by host membranes in a tunnel structure progressively remodelled with each invaded cell

In all invasion events examined, invading hyphae were fully enveloped by host membranes on all sides (except at contact points with glycogen stores (see below)) so that hyphae were always found within a continuous tunnel structure that could extend through successively invaded host cells. In contrast, non-invading filaments observed at the Caco-2 monolayer surface were not associated with any external membranes. Careful examination of the data revealed that tunnel organization is linked to the sequence in which host cells were invaded (see Fig. 4B, Supplementary Movie 11). Invasion into the first host cell in sequence (cell 1) by a filament originating from the surface of the Caco-2 monolayer, was characterized by a tight enveloping of the invading filament by a single host membrane, which in turn was surrounded by an ‘exclusion layer’, that contained no host cellular compartments or organelles (see Fig. 4Ba, Bb and inset (b)). Based on previously published work demonstrating localized actin recruitment at the site of *C. albicans* invasion into Caco-2, and similar structures composed of actin observed during *S. flexneri* invasion, this ‘exclusion layer’ likely represents a region of actin enrichment^14,46^. The ‘exclusion layer’ became progressively thinner as the filament extended through cell 1, and was eventually enveloped by the plasma membrane upon exit from cell 1 (see Fig. 4Bc). Invasion into the second host cell in the sequence (cell 2) started with the enveloping of the structures surrounding the filament in cell 1 by the plasma membrane of cell 2 (see Fig. 4Bd), forming a thicker, clearly visible membrane structure surrounding the hypha further into cell 2 (see Fig. 4Be and inset (e)). This membrane structure persisted throughout cell 2 until the exit from the cell, where another enveloping by the plasma membrane of cell 2 took place (see Fig. 4Bf). The transition between cell 2 and the third cell in the sequence (cell 3) appeared to occur in an identical manner, with an enveloping of the membrane structures seen in cell 2 by the plasma membrane of cell 3, so that two clearly distinguishable thick membrane layers could be observed (see Fig. 4Bg, Bh and inset (h)). Taken together, we observe a progressive remodelling of the tunnel structure surrounding invading filaments which occurs in correspondence to the sequence of invaded cells (see Fig. 4C, Supplementary Movie 12).

### Tunnel ‘Inflation’ lumens are directly linked to host glycogen granule stores

In accordance with our live-cell imaging experiments, we observed local ‘inflations’ of host membranes surrounding invading hyphae in five Caco-2 invasion events acquired by SBF-SEM (see Fig. 4Bi, C, Supplementary Table 2). In four out of the five inflations observed, the inflation lumen contained multiple vesicles with a diameter of around 200 nm approximately matching that described for *C. albicans* hyphal extracellular vesicles (HEVs), though the origin of these vesicles could not be determined^59^. Strikingly, in three of the five inflations observed, host glycogen stores (previously identified by EM in Caco-2^60^) in proximity to the inflation appeared to be disrupted (compared to stores not in the vicinity of inflations) with some glycogen granules found within what appeared to be an opening in the tunnel membranes and within the inflation lumen itself. This opening was found towards the posterior side (away from the hypha tip) of the inflation in all three cases (see Fig. 4D, Supplementary Movie 13, Supplementary Table 2).

In the dataset presented here, a portion of the tunnel inflation was found outside of the host cell, though it was directly linked through a narrow passage to the intracellular inflation. The contact points between tunnel inflations and host glycogen stores may represent points of nutrient uptake from the host by *C. albicans* (see Discussion).

### Membrane layer accumulation around invading hyphae corresponding to the invasion sequence is a hallmark of invasion into Caco-2

**Fig. 5:**
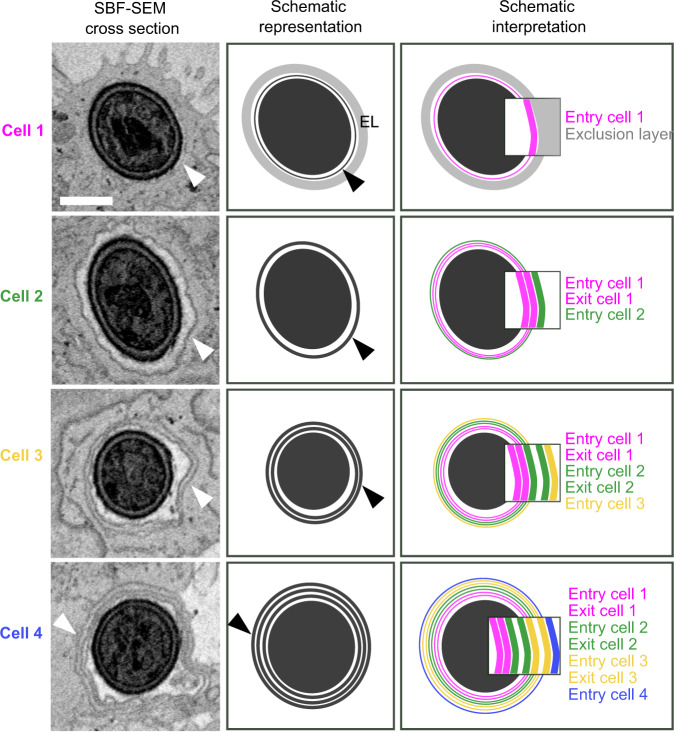
Host membrane organization around a single hypha invading four Caco-2 cells in sequence. Data presented is derived from an SBF-SEM volume containing a single invasion event through four host cells in sequence. A representative section from each invaded host cell in the invasion sequence is presented alongside a schematic representation, illustrating the exclusion layer (EL) and host membrane organization around the hypha in each invaded cell. Arrowheads indicate observed membranes and their corresponding schematic representation. A schematic interpretation is also presented, corresponding to the hypothesis that host membranes extend from each invaded cell into the next host cell in the invasion sequence, resulting in progressive membrane layer accumulation. Insets show in detail inferred membrane organization in accordance with this hypothesis, with each layer derived from a plasma membrane deformed upon hypha entry or exit from each corresponding invaded cell. The presented dataset corresponds to invasion site 9 in Supplementary Table 2 and is presented in Supplementary Movie 14. Scale bar is 1 µm.

Systematic quantification of the organization of the layers surrounding *C. albicans* hyphae in relation to the invasion sequence showed that in every invasion site analysed by SBF-SEM (*n* = 11), the same pattern was reproduced (see Supplementary Table 2): a thin membrane and an exclusion layer in cell 1, a thick membrane in cell 2 and two thick membranes in cell 3. In one striking example, four host cells invaded in sequence by a single hypha was documented by SBF-SEM (see Fig. 5, Supplementary Movie 14). The first three invaded cells showed the above pattern, i.e. thin membrane and exclusion layer in cell 1, thick membrane in cell 2 and two thick membranes in cell 3. In the fourth invaded cell, three thick membranes were observed around the hypha, which is consistent with the accumulating layered structure observed in the three previously invaded cells (see Fig. 5, Schematic representation). Thus, the progressive addition of layers around invading hyphae corresponding to the invasion sequence appears to be a hallmark of Caco-2 invasion, following a reproducible pattern.

One possible interpretation for the origin of this pattern is that each of the thick layers observed around hyphae is in fact composed of multiple membrane bilayers (see Fig. 5, Schematic interpretation). As these thick layers are similar in thickness to the host nuclear envelope (see Fig. 4Bf), it is likely they are composed of at least two membrane bilayers. However, the acquisition parameters used in this study do not allow resolving multiple bilayers within the thick layers surrounding hyphae, as indicated by our inability to resolve the double membrane of the host nuclear envelope. According to this interpretation, the multiple bilayers composing each thick layer are derived from the plasma membrane of previously invaded host cells and that of the currently invaded cell. For example, the thick membrane observed in cell 2 would be derived from the plasma membrane initially deformed upon hyphal entry into cell 1, an additional membrane derived from plasma membrane deformed upon hyphal exit from cell 1, and a third membrane derived from plasma membrane deformed upon hyphal entry into cell 2. We stress that this is only one possible interpretation of the observed data, and that higher resolution approaches as well as functional studies will be required to demonstrate this option or others (see Discussion).

## Discussion

Our analysis of invasion scenarios in HeLa cells showed that both damaging and non-damaging invasion is possible in this cell line, while in Caco-2 cells, invasion is always non-damaging at early time points of infection. Using CM to stain host membranes enveloping invading hyphae, we show that non-damaging invasion in both cell types is always characterized by hyphae traversing host cells within trans-cellular tunnels (TCTs), in a process we term ‘*C. albicans* trans-cellular tunnelling’ (CaTCT). TCTs are also clearly observed in two of the damaging scenarios in HeLa: ‘exit site’ and ‘cell death-associated’ at least until the point of membrane breaching or host cell death, indicating that in these scenarios tunnelling also takes place, though it eventually results in damage. In the rest of HeLa invasion scenarios, continuous TCTs were either not observed (‘multiple sites’ scenario) or could not be clearly discerned (‘entry site’ scenario), suggesting that some HeLa invasion scenarios may not be associated with TCTs, though further research is required to address this possibility. Why some invasion events in HeLa result in CaTCT, while others result in damage remains an open question.

We find that host membrane breaching identified via Gal-3 recruitment during HeLa cell invasion, coincides (within the temporal resolution of our setup) or is followed by host cell death in the vast majority of invasion events studied. Thus, membrane breaching of this type is largely ‘catastrophic’ to host cells, and therefore not successfully repaired to a degree that allows host survival in most cases. Still, a functional role for host membrane repair mechanisms during invasion cannot be ruled out, as these could be employed to successfully repair either membrane breaches too small to be detected by Gal-3, or larger breaches that do not result in host cell death. Indeed, it has been recently shown that galectin reporters are only sensitive for tracking “advanced” damage of endomembranes, and that early or temporal damage cannot be tracked with this reporter^43^. Furthermore, a recent study has demonstrated that membrane breaches induced by candidalysin in TR146 cells during the first hours of invasion can be repaired via an ESCRT-mediated mechanism^61^. Thus, candidalysin-induced breaches that are subsequently repaired are likely too small to be detected by Gal-3 recruitment, as has been shown for breaches formed by the bacterial toxin Listeriolysin O (LLO) produced by *Listeria monocytogenes*^43^. Indeed, as the action of candidalysin requires toxin accumulation inside the invasion pocket, it is unlikely that damage detected during the “entry site” invasion scenario is caused by candidalysin, as the invasion pocket is barely formed at this stage^22^. Rather, the damage detected in this study is likely, at least in part, to be the result of other factors such as the mechanical strain on the host plasma membrane caused by hyphal extension. It has recently been shown that damage caused by mechanical strain during invasion may be repaired via lysosome exocytosis, mediated by the host autophagy machinery and by galectin-3 recruitment itself ^51,61^. Overall, we cannot exclude the presence of host membrane damage not detected by Gal-3, as well as a role for host repair mechanisms, during both damaging and non-damaging invasion scenarios. The precise nature of damage as well as the role of host repair mechanisms during invasion remains to be explored.

Invasion in both HeLa and Caco-2 was not altered by treatment with Cytochalasin D, a drug known to inhibit invasion via induced endocytosis. Thus, in our models, CaTCT as well as damaging invasion in HeLa is distinct from invasion by induced endocytosis, at least according to this distinguishing criterion. This result is consistent with studies suggesting that induced endocytosis does not play a major role in HeLa and Caco-2 invasion, though endocytosis was directly monitored in HeLa during *C. albicans* invasion, where it has been suggested to occur via an E-cadherin-independent mechanism^14,29,30,51^. Whether or not CaTCT is simply active penetration remains an open question. While the current view holds that only two mechanisms (induced endocytosis and active penetration) drive *C. albicans* intracellular epithelial invasion, our work, performed at the single cell level, has revealed several invasive lifestyles (CaTCT, damaging invasion associated with TCTs, and damaging invasion that may not be associated with TCTs) that may be manifestations of a single invasion mechanism, or alternatively, may be mechanistically distinct from one another at the molecular level. Addressing this question requires a systematic investigation of the fungal and host molecular mechanisms that underly CaTCT and damaging invasion scenarios. Further work is also required for understanding the interface between these lifestyles and induced endocytosis, performed in models where induced endocytosis is known to occur, such as oral and vaginal epithelial cell lines or endothelial cell lines. Ultimately, the role of CaTCT in *C. albicans* infection should be examined in more physiologically relevant models^62^.

CaTCT appears to be a complex process, involving significant re-organization of host membranes and compartments during hyphal extension which occurs without host membrane breaching, even as hyphae extend through multiple invaded cells over several hours. In Caco-2, CaTCT characterizes all invasion events until at least 9 h post infection. Host membrane breaching and cell death are observed at later time points (around 24 h post-infection), in agreement with published studies^11,14^. Thus, CaTCT is a phenomenon of early time points of infection, after which a transition towards damaging invasion takes place. This later phase is likely dominated by the action of candidalysin, as *C. albicans* mutants not expressing candidalysin do not damage host cells even at later time points, despite their ability to invade normally^18^.

**Fig. 6:**
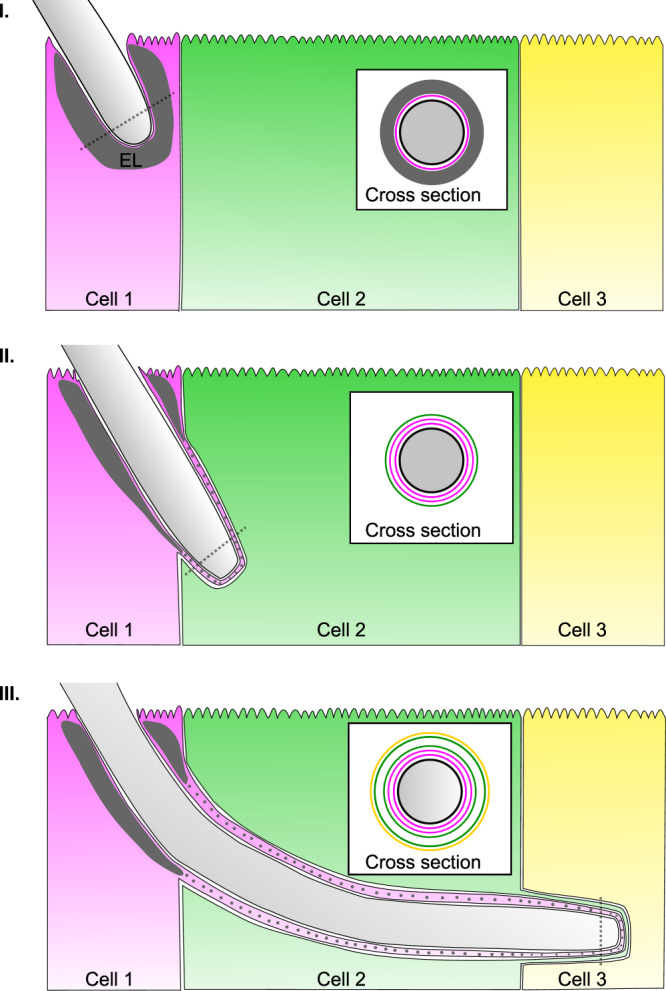
Model of *C. albicans* trans-cellular tunnelling (CaTCT). A model based on the hypothesis that host membranes extend from each invaded cell into the next host cell in the invasion sequence, resulting in progressive membrane layer accumulation. Alternatively, additional membrane layers may be recruited from internal host endocytic compartments or formed de novo in each invaded cell in correspondence with the invasion sequence, though we reason these are less likely possibilities (see Discussion). These latter models are not presented. **I** Invasion into the first cell in the invasion sequence (cell 1) is characterized by tight enveloping by the cell 1 plasma membrane as well as an ‘exclusion layer’ (EL), likely containing actin derived from the apical actin cortex. **II** At the onset of invasion into the second cell in the sequence (cell 2), the structures surrounding the hypha in cell 1, i.e. cell 1 plasma membrane (added during cell 1 entry), and another cell 1 plasma membrane (added during cell 1 exit), are enveloped by the plasma membrane of cell 2. Parts of the exclusion layer in cell 1 may possibly also extend into the TCT structure (grey dots). **III** Invasion into cell 3 proceeds in a similar manner, with the structures surrounding the hypha in cell 2 enveloped by the plasma membrane of cell 3. Cross-section views (derived from dotted lines) show the membrane layers surrounding the hypha cell wall (black), with the exclusion layer in dark grey, and each membrane layer coloured according to its cell of origin, i.e. magenta- cell 1, green- cell 2, yellow- cell 3. Additional actin layers derived from non-apical cortical actin may also be present within the TCT structure, but they were not illustrated for clarity. The model presented applies to non-damaging invasion, and does not account for scenarios where host membrane breaching occurs.

Based strictly on structural considerations derived from fluorescence microscopy and SBF-SEM, we propose that CaTCT proceeds through the following sequence of events (see Fig. 6): invasion into the first host cell begins with a tight enveloping of the host plasma membrane around the extending hypha, which in turn is enveloped by an exclusion layer, likely derived from the host’s apical actin cortex^63^. These two layers constitute the TCT during invasion through the first host cell in the invasion sequence (see Fig. 6I). Exiting the first cell and entering the second cell in the invasion sequence adds two more membranes to the TCT structure, the first derived from the first cell’s plasma membrane upon hypha exit, and the second from the second cell’s plasma membrane upon hypha entry (see Fig. 6II). Thus, within the second invaded host cell the TCT is composed of three membrane layers (two extending from the first invaded cell, and one extending from the second invaded cell), perhaps along with remnants of the exclusion layer found between the two membranes derived from the first invaded cell. This sequence is repeated during invasion into the third cell in the invasion sequence, with the TCT composed of five membrane layers during hyphal extension within the third cell (see Fig. 6III), and so on, until a transition to damaging invasion occurs. Alternatively, the progressive addition of membrane layers to the TCT structure within each invaded host cell in the invasion sequence could be due to recruitment of internal host compartments (such as ER or Golgi) or from membranes synthesized de novo within each invaded cell. While we did not directly differentiate between these two models in our study, we find the latter model less plausible, as it requires each host cell in the invasion sequence to employ different membrane recruitment or synthesis patterns according to its place in the invasion sequence. A combination of both models, relying on contributions to the TCT structure both from previously invaded host cells’ plasma membrane and recruitment of internal membranes, is also possible. Higher resolution approaches, functional assays directly examining the transfer of membranes from one host cell to another within the invasion sequence, as well as studies of the different endocytic compartments possibly implicated in invasion are required to further examine these proposed models. Overall, CaTCT stands in stark contrast to cell-to-cell spreading of actin-motile invasive bacteria like *L. monocytogenes* and *S. flexneri* that must induce host membrane breaching followed by ‘escape’ into the host cytosol in every invaded cell before transitioning to its neighbour^64^.

Remarkably, an exclusion layer in the first invaded Caco-2 cell was observed in all SBF-SEM datasets quantified. Actin re-organization and recruitment associated with *C. albicans* invasion is well documented, and has been identified in different cell types, including Caco-2^14,36^. In published data, actin recruitment is found at or close to the site of hypha insertion into the Caco-2 layer, and is not observed further along the hyphae inserted within the layer^14^. A structurally similar exclusion layer composed of actin has been observed during the early stages of *S. flexneri* invasion into epithelial cells^46,65^. These observations correspond well with the thickness of the exclusion layer identified in this study, which is found within the first invaded cell. While it is likely the exclusion layer is derived from the host apical actin cortex to some degree, the mechanism and molecular factors underlying its formation and extension into the host remain to be explored. As CaTCT may require extensive host plasma membrane stretching over several host cells, the exclusion layer could provide structural support to the TCT structure, though further research is required to address this possibility^66,67^. It is possible that *C. albicans* induced TCTs share some common traits with tunnelling nanotubes (TNTs), defined as open membranous channels between connected cells, which can stretch up to several hundred microns and contain actin within^68^. Indeed, TNT-like structures extending away from invaded HeLa into the region between cells were observed in this study.

We hypothesize that TCT ‘inflations’ found in direct contact with disrupted host glycogen stores may represent sites of nutrient uptake by *C. albicans* during CaTCT. *C. albicans* access to the host’s nutrient rich cytosol during CaTCT is likely highly restricted, due to hyphae being enveloped in multi-layered TCTs. Host glycogen stores could provide an ideal site for nutrient uptake that can be readily utilized by *C. albicans*, though further research is required to determine the role of TCT ‘inflations’ in CaTCT^69^.

As CaTCT occurs without direct contact between invading hyphae and the host cytosol (with the exclusion of the inflation-glycogen store interface) and without host damage, from an immunological standpoint, CaTCT may be perceived by host cells as an entirely extracellular process. Thus, both innate immune recognition of *C. albicans* pathogen-associated molecular patterns (PAMPs) and the activation of epithelial damage-associated molecular patterns (DAMPs) may be similar to those triggered by non-invading adherent *C. albicans* hyphae^23,70–72^. This hypothesis is supported by a study showing that *C. albicans* infection of Caco-2 produces only low levels of both host cellular stress responses and proinflammatory cytokines during the first hours of infection^16^. Overall, CaTCT may represent a ‘commensal-invasive’ lifestyle that does not damage host cells and limits host-pathogen interactions, therefore triggering only a low-level immune response.

Trans-cellular migration has been described in the context of various healthy and pathogenic processes including trans-cellular diapedesis, in which leukocytes travel through endothelial cells in order to enter or leave blood vessels, malaria sporozoites traversal of hepatocytes via transient vacuoles, and invasion by the blast fungus *Magnaporthe grisea* hyphae into rice leaf cells^73–75^. Hyphae of the fungal pathogen *Aspergillus fumigatus* have been reported to traverse human pulmonary epithelium without disrupting host membrane integrity using a tunnel supported by actin, suggesting that trans-cellular tunnelling is perhaps a common trait among different fungal pathogens^76,77^.

In conclusion, this study provides a detailed view of the invasive lifestyles of *C. albicans* during the early stages of infection of epithelial cells, entailing either host membrane breaching or non-damaging host cell traversal. The trans-cellular tunnelling mechanism described here sheds light on long standing questions regarding the precise nature of invasion by this fungal pathogen.

## Methods

### *Candida albicans* strains and growth conditions

*C. albicans* BWP17 prototroph wild-type strain (*ura3Δ::λimm434/ura3Δ::λimm434 his1::hisG/HIS1::his1::hisG arg4::hisG/URA3::ARG4::arg4::hisG*)^78^ or *C. albicans* BWP17 strain expressing GFPγ-CtRac1 at the plasma membrane (*ura3Δ::λimm434/ura3Δ::λimm434 his1Δ::hisG/his1Δ::his arg4::hisG/arg4Δ::hisG RPS1::ARG4-ADH1-GFP*γ*-CtRac1*)^79,80^ (kindly provided by Robert Arkowitz, University of Nice-Sophia Antipolis, France) were used in all assays as indicated. Strains were routinely grown on YPD liquid/agar (1% yeast extract, 2% peptone, 2% D-glucose with or without 2% agar) supplemented with 80 µg/mL of uridine at 30 °C.

### Epithelial cell lines culture

A HeLa cell line stably expressing eGFP-galectin-3 (Gal-3) was generated by Patricia Latour Lambert (Institute Pasteur, Paris, France) using the pLenti6/V5 Directional TOPO Cloning Kit (Invitrogen) with the coding sequences from the pEGFP-galectin-3 plasmid^42,81^. All experiments on HeLa were performed using the HeLa-Gal-3 cell line. Caco-2 cell line subclone C2BBe1 stably expressing eGFP-galectin-3 (Gal-3) was generated as previously described^54^. Cell lines were kindly provided by P. Latour Lambert and Jost Enninga (Institute Pasteur, Paris, France). Caco-2 not expressing Gal-3 were purchased from Sigma (86010202). All cells were routinely cultured in Dulbecco’s modified Eagle’s medium (DMEM) (Gibco-Invitrogen) supplemented with 10% v/v fetal bovine calf serum (Gibco-Invitrogen) at 37 °C, 5% CO_2_. Live-cell imaging experiments were performed in optically transparent EM medium (120 mM NaCl, 7 mM KCl,1.8 mM CaCl_2_, 0.8 mM MgCl_2_, 5 mM glucose and 25 mM Hepes at pH 7.3)^46^ supplemented with 0.2 g/L of amino acids (MP Biomedicals SC Amino Acids Mix) and 80 µg/mL of uridine.

### Infection procedure

For all experiments, epithelial cells were plated in a µ-Plate 96 Well Black suitable for microscopy (Ibidi, Cat. 89626). 1.8-3.5 × 10^4^ HeLa cells were plated 1 to 2 days before infection and were infected at 90% confluency. 1-1.5 × 10^4^ Caco-2 cells were plated 6 to 7 days before infection and were infected 24 hours after reaching confluency^53^. Caco-2 polarization was confirmed by the presence of microvilli at the apical surface observed by EM. Prior to infection, the host cells were washed two times with PBS. To label the host cell plasma membrane and endocytic compartment, CellMask Deep Red Plasma Membrane Stain or CellMask Orange Plasma Membrane Stain (ThermoFisher) was used at 2.5 µg/mL working concentration. Epithelial cells were incubated in the dark at 37 °C for 10 minutes in EM medium, and washed one time with PBS. *C. albicans* cells were always added after epithelial CM staining and washes, and therefore were never in direct contact with this stain. For cytochalasin inhibition experiments, host cells were treated with 0.5 µM of cytochalasin D at 37 °C for 45 min in EM medium, and washed one time with PBS. *C. albicans* strains were cultured overnight in liquid YPD medium supplemented with 80 µg/mL of uridine at 30 °C, 200 rpm shaking, followed by a back dilution at 1:100 into fresh YPD medium supplemented with 80 µg/mL of uridine and then grown to exponential phase at 30 °C, 200 rpm shaking. To prevent yeast cells from clumping together, the culture was subjected to mild sonication on ice for 6 cycles of 15 seconds on and 30 seconds off ^82^, at power setting 15% using the Fisherbrand Model 50 Sonic Dismembrator (Fisher Scientific) with a standard 1/8” diameter microtip. Sonicated yeast cells were counted with a hemacytometer, diluted in EM medium and added to epithelial cells at a multiplicity of infection (MOI) of 0.1 (7 × 10^3^ cell per well), followed by 15 min of incubation to allow initial adherence of yeast cells to host cells. For Caco-2 experiments, live-cell imaging was started 2 hours post-infection to maximize imaging of invasion events. All infection experiments were performed in EM medium without fetal bovine calf serum.

### Live-cell imaging

Live-cell imaging was performed using a fully automated inverted wide-field AxioObserver microscope (Zeiss) equipped with Colibri 7 LED illumination (Zeiss), a Hamamatsu camera (ORCA-Flash4.0 V3), a Piezo stage (Prior Scientific, NanoScanZ 100), an environmental chamber with temperature control set to 37 °C and a Definite Focus 2 device (Zeiss) used to counter focus drift. For initial investigations into HeLa, a 40X air objective was used (Plan-Neofluar NA 0.6) (see Fig. 1). For all other live-cell imaging experiments a 40X oil immersion objective (Plan-Apochromat NA 1.4) was used. Data was acquired using ZEN 2.6 pro software (Zeiss). Movies were recorded over 7 h (such that total time post infection at the end of the acquisition was 7 h for HeLa and 9 h for Caco-2). Every 10 min, a z-stack of 15 planes with 0.94 µm z steps (40X air objective) or a z-stack of 30 planes with 0.37 µm z steps (40X oil objective) was acquired sequentially in two fluorescent channels, with a single plane acquired in phase. Gal-3 was excited with a 475 nm LED and detected with a 525 nm filter. The CellMask Orange Plasma Membrane Stain was excited with a 555 nm LED and detected with a 605 nm filter and the CellMask Deep Red Plasma Membrane Stain was excited with a 630 nm LED and detected with a 690 nm filter. The BFC stage was defined as t = 0 min for every invasion event presented in the figures.

### Serial block face-scanning electron microscopy

For Serial Block Face-Scanning Electron Microscopy (SBF-SEM), Caco-2 (not expressing Gal-3) were infected by *C. albicans* BWP17 wild-type strain for 6 h at MOI 2, then fixed in 3% PFA, 1% glutaraldehyde during 1 h at room temperature. To increase conductivity of the sample in the microscope, the cell monolayer was covered with a thin layer of 10% gelatine containing 1% of Bovine Serum Albumine (BSA). Samples were then prepared for SBF-SEM (NCMIR protocol^83^) as follows: cells were post-fixed for 1 h in a reduced osmium solution containing 1% osmium tetroxide, 1.5% potassium ferrocyanide in PBS, followed by incubation with a 1% thiocarbohydrazide in water for 20 min. Subsequently, samples were stained with 2% OsO_4_ in water for 30 min, followed by 1% aqueous uranyl acetate at 4 °C overnight. Cell monolayers were then subjected to en bloc Walton’s lead aspartate staining^84^, and placed in a 60 °C oven for 30 min. Samples were then dehydrated in graded concentrations of ethanol for 10 min in each step. The samples were infiltrated with 30% agar low viscosity resin (Agar Scientific Ltd, UK) in ethanol, for 1 h, 50% resin for 2 h and 100% resin overnight. The resin was then changed and the cells were further incubated during 3 h, prior to inclusion in upside down capsules and polymerization for 18 h at 60 °C. The polymerized blocks were mounted onto aluminium stubs for SBF imaging (FEI Microtome 8 mm SEM Stub, Agar Scientific), with two-part conduction silver epoxy kit (EMS, 12642-14). For imaging, samples on aluminum stubs were trimmed using an ultramicrotome and inserted into a TeneoVS SEM (ThermoFisher). Acquisitions were performed with a beam energy of 3 kV, 200 pA current, in LowVac mode at 40 Pa, a dwell time of 1 µs per pixel at 10 nm pixel size. Sections of 100 nm were serially cut between images. Overall, 11 different datasets were acquired, containing 11 invasion events with hyphae entirely or almost entirely within the acquisition volume, and 30 invasion events containing hyphae partially within the acquisition volume.

### Western Blot and immunoprecipitation

HeLa and Caco-2 cells expressing or not eGFP-galectin-3 were lysed for 30 min in RIPA-buffer (30 mM Tris-HCl pH 7.4, 150 mM NaCl, 1% Triton X-100, 0.5% sodium deoxycolate, 0.1% SDS, 1 mM EDTA), supplemented with a protease inhibitor cocktail (Sigma-Aldrich). After removal of the insoluble material by centrifugation at 12,000 g, the lysates were either mixed with a concentrated Laemmli buffer or incubated for 3 h at 4 °C with GFP-Trap beads (Chromotek) to immunoprecipitate eGFP-galectin-3. The proteins in the lysates and the immunoprecipitates were separated by SDS-PAGE followed by a transfer to a polyvinylidene difluoride (PVDF) membrane. The membrane was blocked in Tris-buffered saline buffer containing 5% bovine serum albumin and 0.1% Tween 20 for 1 h at room temperature, before incubation with appropriate combinations of primary and secondary antibodies and visualization using the Li-cor Odyssey Imaging System. The primary antibodies used were a mouse anti-galectin-3 mAb (1:1000, sc-32790, Santa Cruz Biotechnology), a rabbit polyclonal anti-GFP (1:2000, Proteintech) and a mouse anti-GAPDH mAb (1:2000, Proteintech). Uncropped and unprocessed scans of the blot are included in the Source Data file.

### Differential staining invasion assay

HeLa expressing Gal-3 or Caco-2 not expressing Gal-3 were fixed with 4% PFA for 10 min at room temperature 4 h or 6 h post infection, respectively. Infected Hela were washed three times with PBS and the fungal cells were first labelled with 10 µg/mL of concanavalin-A coupled with tetramethylrhodamine (Invitrogen) during 20 minutes at room temperature, hence labelling non-invasive fungal cells and the external parts of invasive fungal cells as previously described^30^. For fixed Caco-2, a rabbit anti-*C. albicans* polyclonal antibody at 2,5 µg/mL (OriGene) counterstained with a secondary goat anti-rabbit IgG conjugated with Alexa Fluor 555 at 0.4 µg/mL (Invitrogen) was used as a first label as previously described^14^. After rinsing three times with PBS, the fungal cells were labelled with 25 µg/mL of Remel BactiDrop Calcofluor White (ThermoFisher) during 20 min at room temperature, labelling non-invasive fungal cells and both external and internal parts of invasive fungal cells as previously described^14,30^. After rinsing 3 times with PBS, the fixed samples were directly imaged under an inverted wide-field microscope AxioObserver (Zeiss) with a 63X oil objective (Plan-Apochromat NA 1.4). Calcofluor white was excited with a 385 nm LED and the signal was detected with a 480 nm filter. Concanavalin-A conjugated with tetramethylrhodamine or the secondary antibody was excited with a 555 nm LED and the signal was detected with a 605 nm filter. Depending on the infection site, a z-stack (mean total range of 14 µm) with 0.25 µm z steps was acquired in four channels, including phase and three fluorescent channels (Gal-3, first label, second label).

### LDH release assay

HeLa cells or Caco-2 cells were infected with *C. albicans* BWP17 wild-type strain at MOI 0.1 (7 × 10^3^ cell per well) and MOI 1 (7 × 10^4^ cell per well), as described above (see Infection procedure). LDH release during infection was measured at 3, 6- and 24-h post-infection using the CyQUANT™ LDH Cytotoxicity Assay Kit (Invitrogen) by following the manufacturer’s instructions. As controls, LDH release was measured in the surrounding medium of epithelial layers incubated with growth medium alone (background cells) and from wells without epithelial layers but seeded with *C. albicans* cells in growth medium (background *C. albicans*). Additionally, the maximal LDH release of epithelial cells was obtained by treatment with 1% Triton X-100 to each well and vigorously disrupting the epithelial layer with a pipette tip 1 h before each measured timepoint. The cytotoxicity percentage of epithelial cells infected with *C. albicans* BWP17 wild-type strain was calculated as previously described^14^:

$$\% {{{{{\rm{cytotoxicity}}}}}}=\frac{({\mbox{Experimental LDH release - background cells - background}}\;C.\,albicans)}{\mbox{(Maximal LDH release - background cells)}}$$

All experiments were performed in triplicate for each condition and repeated three times. Data are presented in Supplementary Table 1 as mean ± standard deviation (SD).

### Propidium Iodide cell death assay

Prior to infection, HeLa expressing Gal-3 or Caco-2 expressing Gal-3 were washed two times with PBS. To label the host cell plasma membrane and endocytic compartment, CellMask Deep Red Plasma Membrane Stain (ThermoFisher) was used at 2.5 µg/mL working concentration. Epithelial cells were incubated in the dark at 37 °C for 10 min in EM medium, and washed one time with PBS. Subsequently, host cells were incubated with propidium iodide (PI) (Invitrogen) at 500 µg/mL for HeLa expressing Gal-3 or 2.5 mg/mL for Caco-2 expressing Gal-3 in EM medium during 10 min at room temperature. After removing the propidium iodide solution, the host cells were infected with *C. albicans* BWP17 wild-type strain at MOI 0.1 (7 × 10^3^ cell per well) as described above (see Infection procedure). Imaging was started 15 min post-infection for Hela with images acquired every 10 min for 7 h. For Caco-2 late time points study, imaging was started at 19 h post-infection with images acquired every 10 min for 10 h. For HeLa cell invasion datasets, a total number of 358 invasion events were recorded in five independent replicates. The Chi-square (χ2) test was used to assess the homogeneity in the size of distribution of invasion scenarios among the five independent replicates, with a *p* value threshold of 0.05 (*p* value = 0.025). To quantify the host cell death timing, cell death was assigned to an invasion stage if it occurred within 30 min (three frames) of that stage’s onset when PI was observed. We considered host cells alive at the end of invasion if no host cell death was observed at least 90 min after the SC stage. Host cells that were alive after SC but were not recorded for an additional 90 min due to the end of the live imaging session were discarded for the purpose of cell death quantification (98 invasion events discarded). In total, 260 invasion events were used for cell death quantification.

### Image processing and analysis

Data acquired by fluorescent microscopy were analysed using ZEN 2.6 pro software (Zeiss, blue edition) and Fiji^85^. Data acquired by SBF-SEM were processed using Fiji and Amira (ThermoFisher). Data alignment and manual segmentation were performed using Amira. Figures were prepared using Inkscape, with auto-rendering of images in Fig. 5, and pixelated rendering of images in all other figures.

### Quantifications, statistical analysis and reproducibility

For HeLa invasion studies, a total number of 629 invasion events were recorded out of six independent replicates. The Chi-square (χ2) test was used to assess the homogeneity in the size of distribution of invasion scenarios among the six independent replicates, with a *p* value threshold of 0.05 (*p* value < 0.0001). For Caco-2 invasion studies, a total number of 153 invasion events were recorded in three independent experiments. The cell traversal (CT) time was defined as the time needed for a hypha to extend through one host cell, which was calculated for every invasion event as follows: CT time = time of SC − time of FC. The CT time was calculated only for hyphae that reached the SC stage without host cell death occurring. To compare the CT time between each of the five invasion scenarios in HeLa, statistical differences were assessed with a parametric statistical test, a one-way ANOVA test (multiple *t*-Student test comparison) further corrected with a Tukey’s multiple comparisons with a *p* value threshold of 0.05. To quantify the host cell death timing, cell death was assigned to an invasion stage if it occurred within 30 min (three frames) of that stage’s onset. We considered host cells alive at the end of invasion if no host cell death was observed at least 90 min after the SC stage. Host cells that were alive after SC but were not recorded for an additional 90 min due to the end of the live imaging session were discarded for the purpose of cell death quantification. For cytochalasin D treatment experiments, an identical experimental pipeline as described above was used in three independent experiments. For infected HeLa, a total number of 618 invasion events were counted in four independent replicates (*n* = 311 for untreated cells and *n* = 307 for treated cells). The proportion of each invasion scenario was compared between treated and untreated cells with a non-parametric statistical test, a Mann–Whitney *U*-test, with a *p* value threshold of 0.05 (*p* value = 0.2 for ‘Entry site’, *p* value = 0.6857 for ‘Multiple sites’, *p* value = 0.3428 for ‘Exit site’, *p* value = 0.8857 for ‘Cell death associated’, *p* value = 0.6857 for ‘No Gal-3 recruitment’). For infected Caco-2, a total number of 261 invasion events were counted in three independent replicates (*n* = 126 for untreated cells and *n* = 135 for treated cells). As we identified only a single invasion scenario, the number of invasion events in treated cells was normalized by the number of invasion events in untreated cells, and was compared using a non-parametric statistical test, Wilcoxon signed rank test, with a *p* value threshold of 0.05 (*p* value = 0.75). The difference in HeLa invasion scenario distribution in cytochalasin D treated vs. untreated cells was evaluated using a χ2 test for homogeneity with a *p* value threshold of 0.05. Errors are given as standard deviation if not mentioned otherwise. Statistical analysis was performed using GraphPad Prism 9.1.2. Number of times experiments were repeated independently: Figs. 1: 6, 2: 3, 3A–C: 3, 3D: 3, 4: Samples were prepared one time for SBF-SEM acquisition, followed by three different acquisition sessions. Figure 5: data presented was acquired in the same sessions used for Fig. 4. Supplementary Fig. 1: blot repeated three times. Supplementary Figs. 2A: 6, 2B: 3, 3: 3, 4: 5, 5A: 4, 5B: 3, 6: 3, same dataset used for Fig. 2. Supplementary Fig. 7: representative image of observations made in the datasets used in Fig. 1. Supplementary Fig. 8: 2. Supplementary Tables 1: 3, and 2: quantification of data presented in Figs. 4 and 5.

### SBF-SEM data quantification

11 SBF-SEM datasets containing complete or near complete invasion sites were selected for quantification. Raw data was inverted, and each invasion site was aligned in Fiji using the “Linear stack alignment with SIFT” plugin using Translation mode, followed by visual examination and recording of quantified features. Resolution of all datasets is 10 nm x, y and 100 nm in z. All datasets are available at: 10.5281/zenodo.5776104.

### Reporting summary

Further information on research design is available in the Nature Research Reporting Summary linked to this article.

## Supplementary information

Supplementary information

Reporting Summary

Description of Additional Supplementary Files

Supplementary Movie 1

Supplementary Movie 2

Supplementary Movie 3

Supplementary Movie 4

Supplementary Movie 5

Supplementary Movie 6

Supplementary Movie 7

Supplementary Movie 8

Supplementary Movie 9

Supplementary Movie 10

Supplementary Movie 11

Supplementary Movie 12

Supplementary Movie 13

Supplementary Movie 14

## Data Availability

SBF-SEM datasets generated in this study have been deposited in the Zenodo repository with the identifier 10.5281/zenodo.5776104. Live cell imaging datasets produced in this study are available from the corresponding author upon request due to data size and multi-parametric format. Source data are provided with this paper.

## Source data

Source Data
