# Supplementary material for: Trans-cellular tunnels induced by the fungal pathogen Candida albicans facilitate invasion through successive epithelial cells without host damage
Source: Nat Commun. 2022 Jun 30;13:3781. doi: 10.1038/s41467-022-31237-z (PMC9246882; doi:10.1038/s41467-022-31237-z)
Supplement: Supplementary file 1 — Supplementary information [file 41467_2022_31237_MOESM1_ESM.pdf]

Trans-cellular tunnels induced by the fungal pathogen *Candida albicans* facilitate invasion through successive epithelial cells without host damage

<u>Supplementary Information</u>

Supplementary Figures

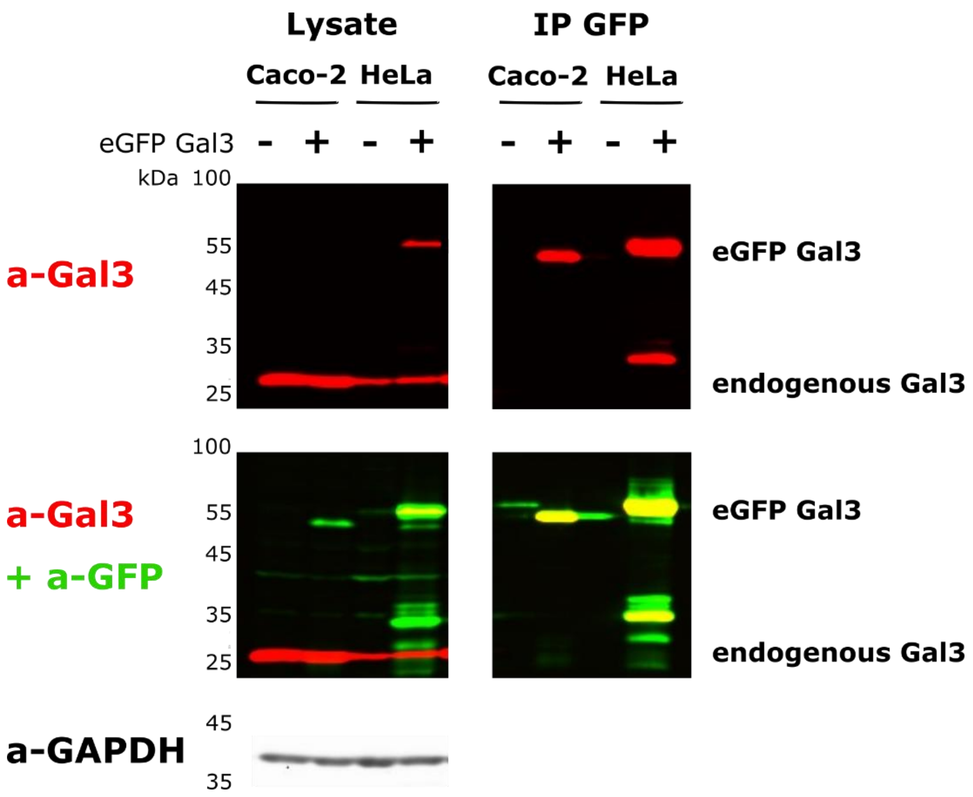

Supplementary Figure 1**. Western blot of total proteins (lysates) and immunoprecipitates (IP-GFP) using GFP-Trap beads show similar or lower expression levels of eGFP-galectin-3 (eGFP Gal3, 55 kDa) compared to endogenous galectin-3 (endogenous Gal3, 27 kDa) in stably expressing HeLa and Caco-2 cell lines respectively**. The membrane was first incubated with an anti-galectin-3 antibody (red, upper panels), followed by an anti-GFP antibody (green, middle panels). The membrane was stained with an anti-GAPDH antibody (37 kDa) as a loading control (black, lower panel). As incubation with an anti-galectin-3 antibody did not yield a visible band at 55kDa in the Caco-2 lysate, we verified the presence of eGFP Gal3 in this cell line using IP-GFP. Source data are provided as a Source Data file.

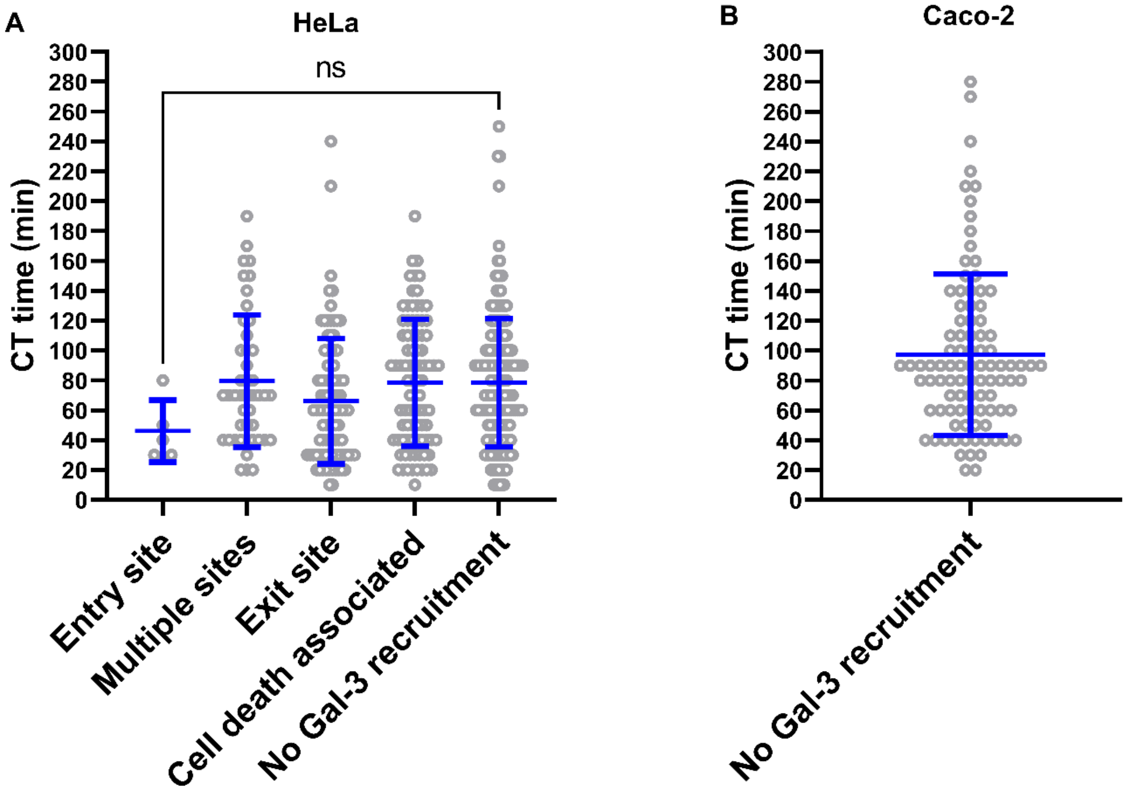

Supplementary Figure 2. **Cell traversal (CT) times in HeLa and Caco-2 cells in each invasion scenario**. For HeLa cells (A), n = 414 invasion events in six independent experiments (n = 5 “Entry site”, n = 45 “Multiple sites”, n = 97 “Exit site”, n = 87 “Cell death associated”, n = 183 “No Gal-3 recruitment”) compared statistically with a one-way ANOVA test (multiple t-Student test comparison) further corrected with a Tukey’s multiple comparisons, p-value < 0.05 (p-value = 0.0706, non-significant (ns)). For Caco-2 cells (B), n = 94. Data are presented as mean values +/- SD. Source data are provided as a Source Data file.

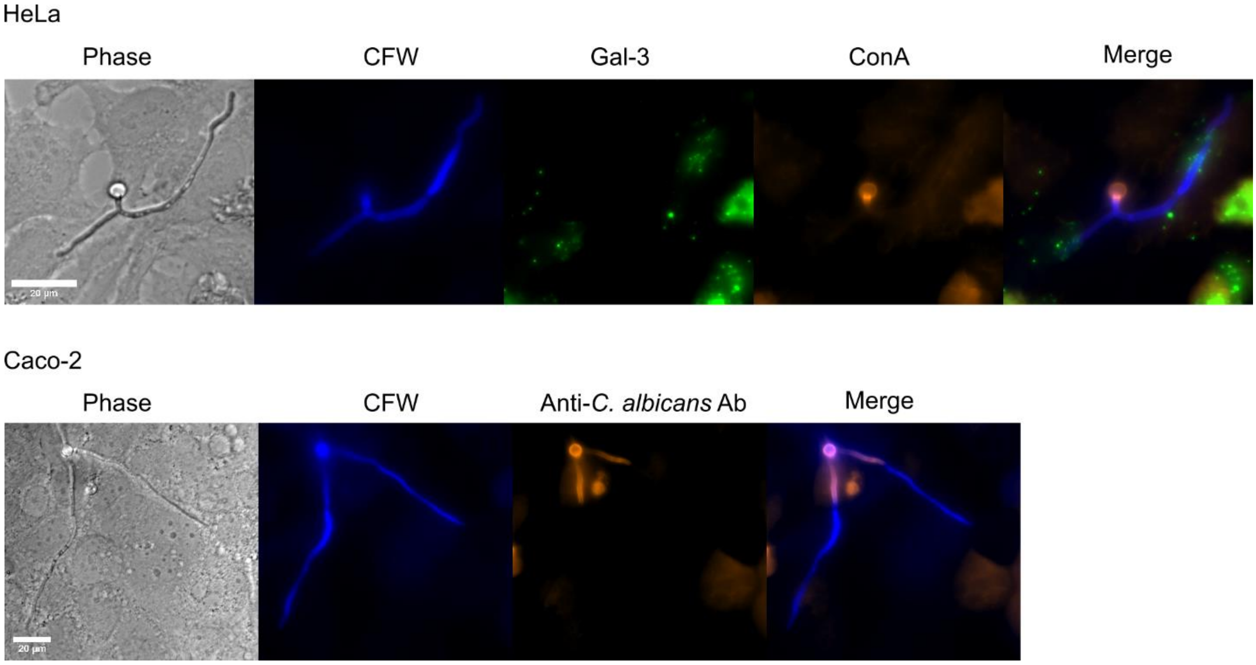

Supplementary Figure 3. **Differential invasion assay in HeLa and Caco-2 cells.** HeLa or Caco-2 infections were fixed at 4 hours and 6 hours post-infection respectively. The culture was stained with Calcofluor White (CFW), labelling entire hyphae and germ cells and concanavalin-A (ConA) or an anti-*C. albicans* antibody (Ab), labelling only the non-internalized part of hyphae. Scale bars are 20 µm.

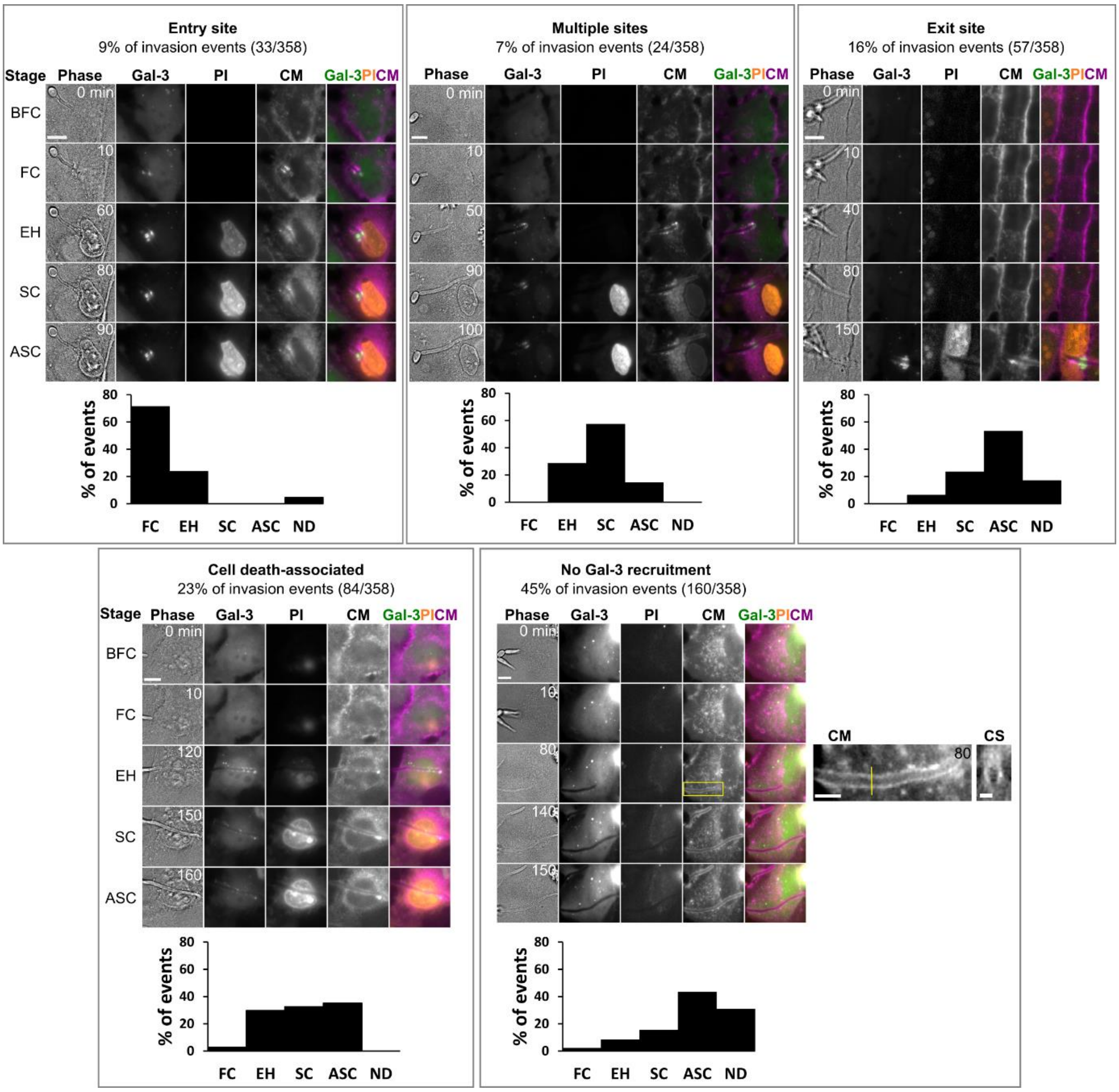

Supplementary Figure 4. ***C. albicans*** **HeLa invasion experiments performed in the presence of the cell death marker propidium iodide (PI)**. A total of n = 358 invasion events were acquired. For each scenario a representative invasion event is presented, divided into the five invasion stages: before first contact with host plasma membrane (BFC), first contact with host plasma membrane (FC), hypha extension within the host (EH), second contact with the host plasma membrane (SC), after second contact with the host plasma membrane (ASC). For each time point a phase image is presented together with a maximum intensity projection of three z-sections in the Gal-3, PI and CellMask (CM) channels, and a composite image showing Gal-3 (green), PI (orange) and CM (magenta) together. An inset (yellow frame) is presented in the 'No Gal-3 recruitment scenario', showing CM labelling in detail. A cross section view (CS, derived from yellow line) is also presented. The distribution of cell death timing as a function of the invasion stage is presented for each scenario. ND stands for no cell death during invasion. Scale bars are 10 µm, 5 µm in the inset and 2 µm in the cross-section view. Source data are provided as a Source Data file.

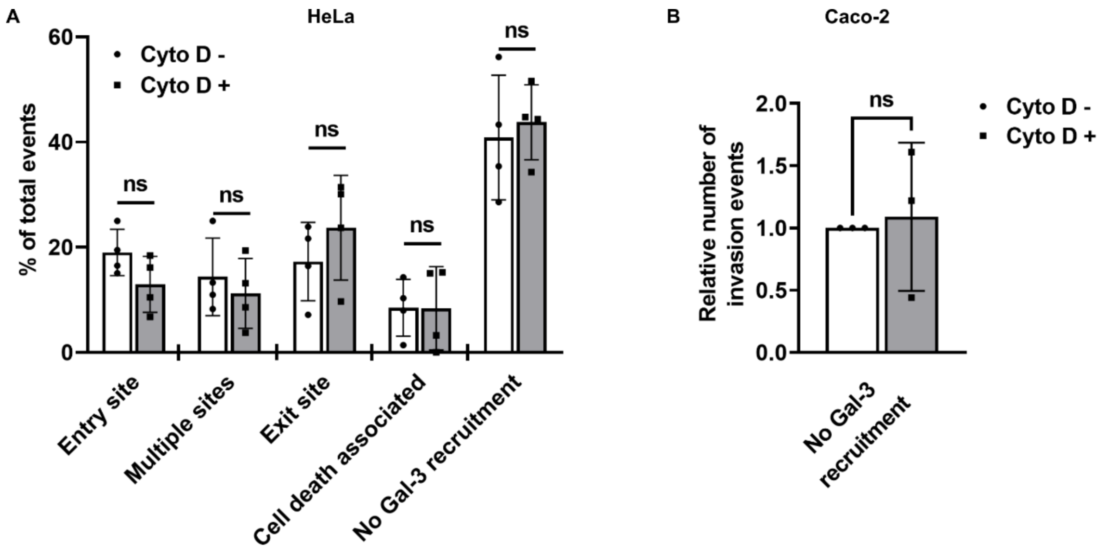

Supplementary Figure 5. **Cytochalasin D treatment does not significantly alter the number of invasion events in HeLa or Caco-2**. (A) HeLa. n = 618 invasion events. The relative proportion of each invasion scenario was compared between treated and untreated cells with a non-parametric statistical test, a Mann-Whitney U-test, with a p-value threshold of 0.05 (p-value = 0.2 for 'Entry site', p-value = 0.6857 for 'Multiple sites', p-value = 0.3428 for 'Exit site', p-value = 0.8857 for 'Cell death associated', p-value = 0.6857 for 'No Gal-3 recruitment' (B) Caco-2, n = 261 invasion events. As we identified only a single invasion scenario, the number of invasion events in treated cells was normalized by the number of invasion events in untreated cells, and was compared using a non-parametric statistical test, Wilcoxon signed rank test, with a p-value threshold of 0.05 (p-value = 0.75). Bars are presented as mean values + SD. Source data are provided as a Source Data file.

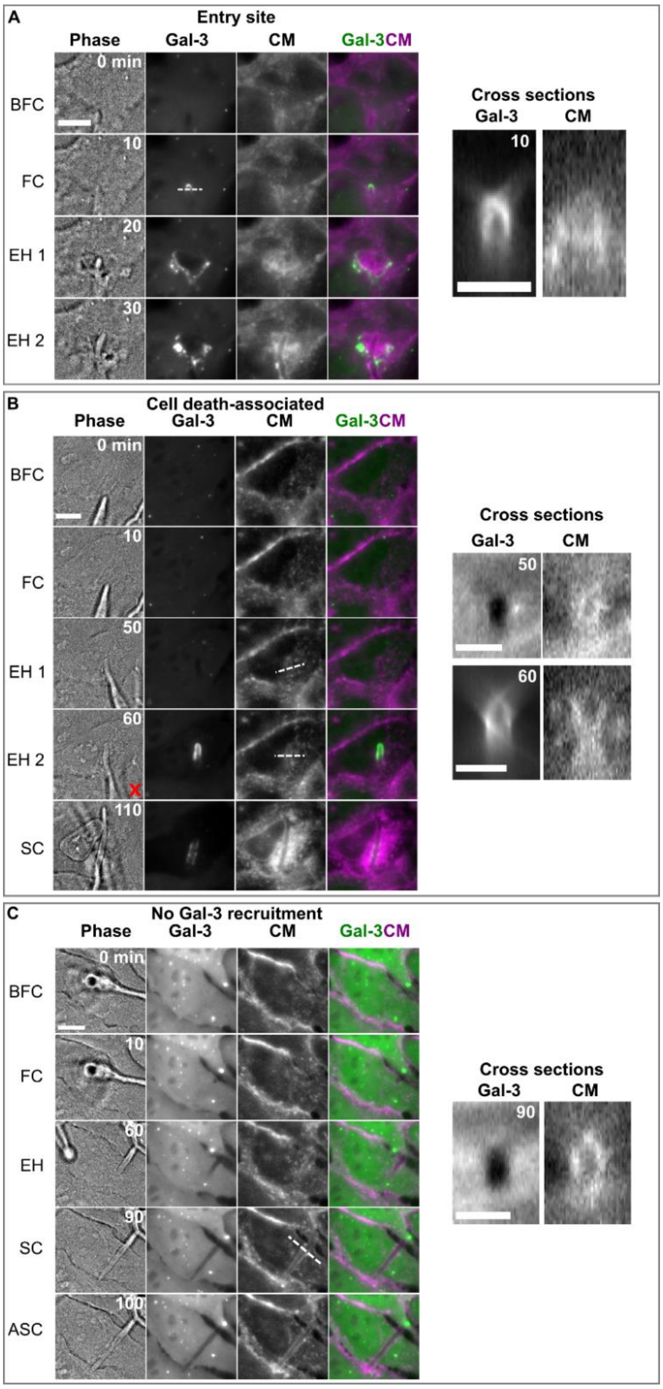

Supplementary Figure 6. **High-resolution live cell imaging of *C. albicans* hyphae invading HeLa cells via the 'entry site', 'cell death-associated' and 'no Gal-3 recruitment' scenarios.** The invasion stages are presented as follows: before first contact (BFC); first contact with the host plasma membrane (FC); extension within the host cell (EH); second contact with the host plasma membrane (SC); after second contact (ASC). Time 0 is set to BFC. For each time point a phase image is presented together with a maximum intensity projection of three z-sections in the Gal-3 and CellMask (CM) channels, and a composite image showing Gal-3 (green) and CM (magenta) together. A cell death event is marked with a red 'x'. Cross-sections derived from the dotted line in the corresponding time point are presented in the Gal-3 and CM channels. Scale bars are 10 µm, and 5 µm in the cross-section views.

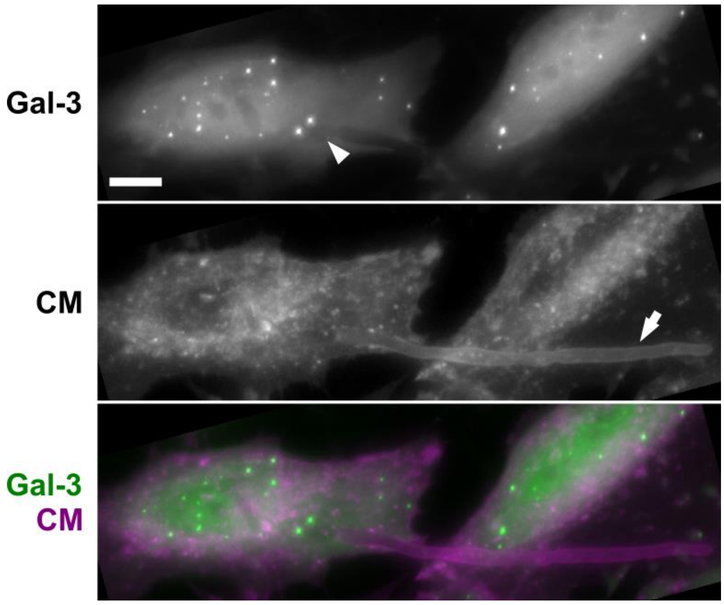

Supplementary figure 7. **CM labelling of a hypha extending into the space between cells after exit from an invaded HeLa cell**. Entry point of hypha is marked by an arrow head. CM labelling is observed along the length of the hypha at a distance from the invaded cell (arrow), suggestive of extensive host membrane stretching. Maximum intensity projection is presented. Scale bar is 10 µm.

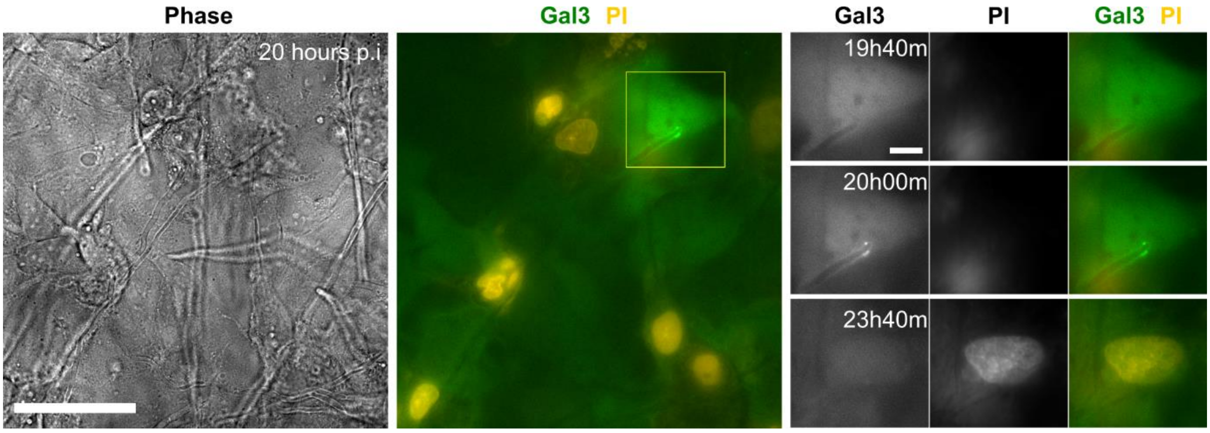

Supplementary Figure 8. **Live cell imaging of WT *C. albicans* invasion into Caco-2 cells expressing Gal-3 and labelled with propidium iodide (PI) at late time points of infection.** Infection of Caco-2 cells was performed in an identical manner to early time point infections (see **Figure 3**). Live cell imaging was launched at 19 hours post-infection (p.i), with images acquired every 10 minutes. At these later times points, Gal-3 recruitment events were easily identifiable, and host cell death (as indicated by PI labelling of the host cell nuclei) was abundant. Inset shows a site of Gal-3 recruitment, with different time points showing the site before Gal-3 recruitment (19h40m), during Gal-3 recruitment (20h00m), and during host cell death (23h40m). Scale bars are 50 µm and 10 µm for inset.

Supplementary Table 1**. LDH release assay†**

Cell line/MOI	3 hours	6 hours	24 hours
HeLa/MOI 0.1	0.00 ± 0.00	0.28 ± 0.40	27.15 ± 7.96
HeLa/MOI 1	0.02 ± 0.03	11.76 ± 0.55	28.65 ± 2.94
Caco-2/MOI 0.1	0.64 ± 0.91	0.85 ± 0.95	29.33 ± 9.91
Caco-2/MOI 1	0.00 ± 0.00	0.05 ± 0.07	35.72 ± 8.63

† LDH release experimental details and calculation of cytotoxicity percentages are described in the methods section. Data are presented as mean ± SD. Source data are provided as a Source Data file.

Supplementary Table 2. **SBF-SEM data quantification**

Invasion site	Number of invaded cells	Membrane organization around hypha†	Presence of exclusion layer	Presence of tunnel inflation	Host cell in the invasion sequence with inflation	Link with host glycogen granules	Vesicles observed within inflation lumen	Point of link between inflation and glycogen store
1*	3	[1] TN, [2] TK, [3] DTK	Yes	Yes	3	Yes	Yes	Inflation posterior**
2	2	[1] TN, [2] TK	Yes, thin	Yes	2	Yes	Yes	Inflation posterior**
3	3	[1] TN, [2] TK, [3] DTK	Yes	Yes	2	Yes	Yes, few	Inflation posterior**
4	3***	[1] TN, [2] TK, [3] DTK	Yes	Yes	2	No	No	
5	2***	[1] TN, [2] TK	Yes, thin	Yes, small	2	No	Yes	
6	1	[1] TN	Yes	No				
7	1	[1] TN	Yes	No				
8	2	[1] TN, [2] TK, split into two layers	Yes, thin	No				
9****	4	[1] TN, [2] TK, [3] DTK, [4] TTK	Yes	No				
10	2***	[1] TN, [2] TK	Yes	No				
11	2***	[1] TN, [2] TK	Yes	No				

† Host cells invaded in sequence are annotated cell [1], [2], [3], [4]. Membrane organization is annotated as: TN- thin membrane, TK- thick membrane, DTK- double thick membrane, TTK- triple thick membrane

* Dataset presented in Figure 4

** The inflation posterior is defined as the edge further away from the hypha tip

*** Partial dataset

**** Dataset presented in Figure 5
