# Supplementary material for: Trans-cellular tunnels induced by the fungal pathogen Candida albicans facilitate invasion through successive epithelial cells without host damage
Source: Nat Commun. 2022 Jun 30;13:3781. doi: 10.1038/s41467-022-31237-z (PMC9246882; doi:10.1038/s41467-022-31237-z)
Supplement: Supplementary file 2 — Reporting Summary [file 41467_2022_31237_MOESM2_ESM.pdf]

nature portfolio

Corresponding author(s): Allon Weiner

Last updated by author(s): May 30, 2022

Reporting Summary

Nature Portfolio wishes to improve the reproducibility of the work that we publish. This form provides structure for consistency and transparency in reporting. For further information on Nature Portfolio policies, see our Editorial Policies and the Editorial Policy Checklist.

Statistics

For all statistical analyses, confirm that the following items are present in the figure legend, table legend, main text, or Methods section.

n/a	Confirmed	
☐	☒	The exact sample size (n) for each experimental group/condition, given as a discrete number and unit of measurement
☐	☒	A statement on whether measurements were taken from distinct samples or whether the same sample was measured repeatedly
☐	☒	The statistical test(s) used AND whether they are one- or two-sided<br>*Only common tests should be described solely by name; describe more complex techniques in the Methods section.*
☒	☐	A description of all covariates tested
☐	☒	A description of any assumptions or corrections, such as tests of normality and adjustment for multiple comparisons
☐	☒	A full description of the statistical parameters including central tendency (e.g. means) or other basic estimates (e.g. regression coefficient) AND variation (e.g. standard deviation) or associated estimates of uncertainty (e.g. confidence intervals)
☐	☒	For null hypothesis testing, the test statistic (e.g. F, t, r) with confidence intervals, effect sizes, degrees of freedom and P value noted<br>*Give P values as exact values whenever suitable.*
☒	☐	For Bayesian analysis, information on the choice of priors and Markov chain Monte Carlo settings
☒	☐	For hierarchical and complex designs, identification of the appropriate level for tests and full reporting of outcomes
☒	☐	Estimates of effect sizes (e.g. Cohen's d, Pearson's r), indicating how they were calculated

Our web collection on statistics for biologists contains articles on many of the points above.

Software and code

Policy information about availability of computer code

Data collection	Light microscopy: ZEN 2.6 pro software (Zeiss, blue edition).
Data analysis	Fiji version 1.53c, Amira version 2020.2., GraphPad Prism 9.1.2

For manuscripts utilizing custom algorithms or software that are central to the research but not yet described in published literature, software must be made available to editors and reviewers. We strongly encourage code deposition in a community repository (e.g. GitHub). See the Nature Portfolio guidelines for submitting code & software for further information.

Data

Policy information about availability of data

All manuscripts must include a data availability statement. This statement should provide the following information, where applicable:

- Accession codes, unique identifiers, or web links for publicly available datasets
- A description of any restrictions on data availability
- For clinical datasets or third party data, please ensure that the statement adheres to our policy

SBF-SEM datasets generated in this study have been deposited in the Zenodo repository with the identifier https://doi.org/10.5281/zenodo.5776104. Live cell imaging datasets produced in this study are available from the corresponding author upon request due to data size and multi-parametric format. Source data are provided with this paper.

Field-specific reporting

Please select the one below that is the best fit for your research. If you are not sure, read the appropriate sections before making your selection.

[x] Life sciences [ ] Behavioural & social sciences [ ] Ecological, evolutionary & environmental sciences

For a reference copy of the document with all sections, see nature.com/documents/nr-reporting-summary-flat.pdf

Life sciences study design

All studies must disclose on these points even when the disclosure is negative.

Sample size	No sample size calculation was performed. Sample sizes were chosen based on the observation and categorization of sufficient invasion events so that the distribution of invasion scenarios within each experimental replicate (in each cell line) was statistically equivalent (statistical test described in materials and methods)
Data exclusions	No data was excluded
Replication	At least three live cell imaging independent experiments were performed for every experimental condition presented. EM sample preparation of Caco-2 invasion sites was performed one time followed by acquisition of 11 datasets. All attempts at replication were successful.
Randomization	Negative. Only cell lines used in the study
Blinding	Negative. Experiments and analysis were performed by the authors without blinding.

Reporting for specific materials, systems and methods

We require information from authors about some types of materials, experimental systems and methods used in many studies. Here, indicate whether each material, system or method listed is relevant to your study. If you are not sure if a list item applies to your research, read the appropriate section before selecting a response.

Materials & experimental systems

n/a	Involved in the study	
[ ]	[x]	Antibodies
[ ]	[x]	Eukaryotic cell lines
[x]	[ ]	Palaeontology and archaeology
[x]	[ ]	Animals and other organisms
[x]	[ ]	Human research participants
[x]	[ ]	Clinical data
[x]	[ ]	Dual use research of concern

Methods

n/a	Involved in the study	
[x]	[ ]	ChIP-seq
[x]	[ ]	Flow cytometry
[x]	[ ]	MRI-based neuroimaging

Antibodies

Antibodies used	Candida albicans Rabbit Polyclonal Antibody (OriGene CAT#: BP1006), mouse anti galectin-3 mAb (1:1000, sc-32790, Santa Cruz Biotechnology), mouse GAPDH Monoclonal antibody (Proteintech, 60004-1-lg, GenBank Accession Number: BC004109, clone 1E6D9)
Validation	Candida albicans Rabbit Polyclonal Antibody validation and citations described in: https://m1.acris-antibodies.com/pdf/BP1006.pdf, mouse anti galectin-3 mAb validation and citations described in: https://datasheets.scbt.com/sc-32790.pdf, mouse GAPDH Monoclonal antibody validation and citations described in https://www.ptglab.com/products/GAPDH-Antibody-60004-1-Ig.htm

Eukaryotic cell lines

Policy information about cell lines

Cell line source(s)	HeLa cell line stably expressing eGFP-galectin-3 and Caco-2 cell line stably expressing eGFP-galectin-3 were obtained from Jost Enninga, Institut Pasteur, Paris, France. Caco-2 cell line was purchased from Sigma (86010202)
Authentication	None of the cell lines used were authenticated
Mycoplasma contamination	All cell lines were tested negative for Mycoplasma contamination using DAPI staining and light microscopy.

Commonly misidentified lines<br>(See ICLAC register)	No commonly misidentified cell lines were used in the study
