# Supplementary material for: Trans-cellular tunnels induced by the fungal pathogen Candida albicans facilitate invasion through successive epithelial cells without host damage
Source: Nat Commun. 2022 Jun 30;13:3781. doi: 10.1038/s41467-022-31237-z (PMC9246882; doi:10.1038/s41467-022-31237-z)
Supplement: Supplementary file 3 — Description of Additional Supplementary Files [file 41467_2022_31237_MOESM3_ESM.pdf]

Title: Supplementary Movie 1
Description: corresponding to figure 1B - HeLa invasion, **‘entry site’ scenario**. From left to right: phase, Gal-3, CM and a Gal-3 (green) and CM (magenta) composite view are presented. Time points match those presented in figure 1B.

Title: Supplementary Movie 2
Description: corresponding to figure 1B - HeLa invasion, **‘multiple sites’ scenario**. From left to right: phase, Gal-3, CM and a Gal-3 (green) and CM (magenta) composite view are presented. Time points match those presented in figure 1B.

Title: Supplementary Movie 3
Description: corresponding to figure 1B - HeLa invasion, **‘exit site’ scenario**. From left to right: phase, Gal-3, CM and a Gal-3 (green) and CM (magenta) composite view are presented. Time points match those presented in figure 1B.

Title: Supplementary Movie 4
Description: corresponding to figure 1B - HeLa invasion, **‘cell death-associated’ scenario**. From left to right: phase, Gal-3, CM and a Gal-3 (green) and CM (magenta) composite view are presented. Time points match those presented in figure 1B.

Title: Supplementary Movie 5
Description: corresponding to figure 1B - HeLa invasion, **‘no Gal-3 recruitment’ scenario**. From left to right: phase, Gal-3, CM and a Gal-3 (green) and CM (magenta) composite view are presented. Time points match those presented in figure 1B.

Title: Supplementary Movie 6
Description: corresponding to figure 2 - HeLa invasion, high resolution. From left to right: phase, Gal-3, CM and a Gal-3 (green) and CM (magenta) composite view are presented. Time points match those presented in figure 2.

Title: Supplementary Movie 7
Description: corresponding to figure 3A - Caco-2 invasion scenario. Top left: phase, top right: Gal-3, bottom left: CM, bottom right: Gal-3 (green) and CM (magenta) composite view. Time points match those presented in figure 3A.

Title: Supplementary Movie 8
Description: corresponding to figure 3B - 3D segmentation of CM labelling during Caco-2 invasion. CM labelling within the entire acquisition volume is segmented in three dimensions (magenta). Background (orange) provides host cell outlines. Time points match those presented in figure 3B, for both XY and XZ views.

Title: Supplementary Movie 9
Description: corresponding to figure 3D - ‘Inflation’ during Caco-2 invasion. From left to right: phase, *C. albicans*, CM and a *C. albicans* (green) and CM (magenta) composite view are presented. Time points match those presented in figure 3D.

Title: Supplementary Movie 10
Description: corresponding to figure 4A - SBF-SEM, overview of invasion site. Data binned to a XY resolution of 20 nm is presented. Axes match those in figure 4A. Slices from the data set are presented in sequence, leading to the apparent “movement” in the movie. Segmentations are as follows: white- *C. albicans* hypha, magenta- host cell 1, green- host cell 2, yellow- host cell 3, brown- host cell nuclei and blue- host glycogen stores.

Title: Supplementary Movie 11
Description: corresponding to figure 4B - SBF-SEM, The organization of host membranes around the hypha in each invaded cell. Data binned to a XY resolution of 20 nm is presented. Segmentation is the same as in Supplementary Movie 10.

Title: Supplementary Movie 12
Description: corresponding to figure 4C - Cross-section view of trans-cellular tunnel. Data binned to a XY resolution of 20 nm is presented via a non-orthogonal slice (in relation to the acquisition volume). Axes match those in figure 4C. Hypha is segmented in white. Trans-cellular tunnel segments are segmented according to their association with host cells the invasion sequence: magenta- host cell 1, green- host cell 2, yellow- host cell 3.

Title: Supplementary Movie 13
Description: corresponding to figure 4D - Direct contact between 'inflation' lumen and host glycogen granules. Data binned to a XY resolution of 20 nm is presented. Segmentation matches that in Supplementary Movie 12 with the addition of host glycogen store in blue. The *C. albicans* Spitzenkörper is also observed.

Title: Supplementary Movie 14
Description: corresponding to figure 5. A hypha invading four host cells in sequence. XY view (acquisition axis) is presented. Scale bar is 5 µm.
